# Arctic Puffin Optimization Algorithm Integrating Opposition-Based Learning and Differential Evolution with Engineering Applications

**DOI:** 10.3390/biomimetics10110767

**Published:** 2025-11-12

**Authors:** Yating Zhu, Tinghua Wang, Ning Zhao

**Affiliations:** Key Laboratory of Data Science and Artificial Intelligence of Jiangxi Education Institutes, Gannan Normal University, Ganzhou 341000, China; 1230740003@gnnu.edu.cn (Y.Z.);

**Keywords:** arctic puffin optimization algorithm, mirror opposition-based learning mechanism, dynamic differential evolutionary strategy, engineering optimization design problems

## Abstract

The Arctic Puffin Optimization (APO) algorithm, proposed in 2024, is a swarm intelligence optimization. Similar to other swarm intelligence optimization algorithms, it suffers from issues such as slow convergence in the early stage, being easy to fall into local optima, and insufficient balance between exploration and exploitation. To address these limitations, an improved APO (IAPO) algorithm incorporating multiple strategies is proposed. Firstly, a mirror opposition-based learning mechanism is introduced to expand the search scope, improving the efficiency of searching for the optimal solution, which enhances the algorithm’s convergence accuracy and optimization speed. Secondly, a dynamic differential evolution strategy with adaptive parameters is integrated to improve the algorithm’s ability to escape local optima and achieve precise optimization. Comparative experimental results between IAPO and eight other optimization algorithms on 20 benchmark functions, as well as CEC2019 and CEC2022 test functions, show that IAPO achieves higher accuracy, faster convergence, and superior robustness, securing first-place average rankings of 1.35, 1.30, 1.25, and 1.08 on the 20 benchmark functions, CEC 2019, 10- and 20-dimensional CEC 2022 test sets, respectively. Finally, simulation experiments were conducted on three engineering optimization design problems. IAPO achieved optimal values of 5.2559 × 10^−1^, 1.09 × 10^3^, and 1.49 × 10^4^ for these engineering problems, ranking first in all cases. This further validates the effectiveness and practicality of the IAPO algorithm.

## 1. Introduction

Swarm intelligence algorithms are meta-heuristic algorithms that mimic the collective behavior of natural biological populations to address complex optimization problems [1,2,3,4]. The core idea is to achieve intelligent overall behavior through simple rules and local information exchange among individuals. In recent years, scholars have developed a range of swarm intelligence algorithms, drawing inspiration from observing and simulating the living habits of organisms. For example, the Slime Mould Algorithm (SMA) [5] mimics the foraging behavior of slime molds and utilizes adaptive weighting to adjust its search direction, rendering it suitable for high-dimensional and multi-modal optimization. However, the algorithm tends to converge slowly and often becomes trapped in local optima. However, it tends to become trapped in local optima and exhibits slow convergence. The Tunicate Swarm Algorithm (TSA) [6] simulates the jet propulsion and group foraging behavior of tunicates, enhancing global exploration through a conflict avoidance mechanism. Nonetheless, it involves multiple parameters, which may lead to performance degradation and poor adaptability in high-dimensional settings. The Snake Optimizer (SO) [7] employs a male–female dual-population mechanism to increase population diversity, but its convergence accuracy is limited. The Particle Swarm Optimization (PSO) algorithm [8] features a simple principle and fast convergence, but it is prone to getting stuck in local optima and is sensitive to parameter settings. The Arctic Puffin Optimization (APO) algorithm [9] employs a versatile multi-stage strategy that integrates behaviors of both aerial flight and underwater foraging to better adapt to complex environments. However, the common challenges are the insufficiently smooth transition between the exploration and exploitation phases and complex parameter adjustment. The Differentiated Creative Search (DCS) algorithm [10] boosts search efficiency via cooperative evolution and multi-strategy fusion, yet it suffers from high implementation complexity and pronounced parameter sensitivity. However, they exhibit weak global exploration capabilities, a tendency to become trapped in local optima, and low convergence accuracy in high-dimensional problems. Given that these algorithms often suffer from issues such as low convergence accuracy and an imbalance between exploration and exploitation, several innovative algorithmic improvements have been proposed. For example, Xia et al. [11] introduced a meta-learning-based alternating minimization method that replaces handcrafted strategies with learned adaptive ones, achieving substantial performance gains. Long et al. [12] proposed an enhanced PSO algorithm, utilizing opposition-based learning to improve initial population quality. Introducing continuous mapping enhances neighborhood search capability, and integrating particle perturbation increases diversity to avoid local optima. Cai et al. [13] proposed a multi-strategy differentiated creative search method, introducing co-evolution to improve the DCS algorithm efficiency. Integrating a composite fitness-distance evaluation enables balanced exploration-exploitation transitions, while linear population size reduction further enhances performance. Guo et al. [14] proposed a “sweep-rotate” gait that enhances planetary rover escape capability from granular terrain. A Bayesian optimization-based escape strategy clarifies variable influences and parameter ranges, improving optimization accuracy. Tian et al. [15] proposed an adaptive improvement method to address the low efficiency of traditional Jump Markov Chain Monte Carlo algorithms. By dynamically adjusting the proposal distribution and employing parallel annealing techniques, they significantly enhanced the efficiency and convergence of curve fitting calculations.

Typical engineering optimization problems exhibit nonlinear, non-differentiable, or multimodal characteristics that hinder the application of traditional gradient-based methods; metaheuristic algorithms emerge as particularly suitable solutions. Their effectiveness lies in their ability to perform a global search without requiring gradient information. Numerous engineering optimization problem models have already been developed. For instance, Kumar et al. [16] addressed classical problems, including tension/compression spring design, which minimizes spring weight by optimizing three design variables, subject to four constraints. Zhou et al. [17] developed a physics-informed neural network framework for fatigue life prediction, which incorporates partial differential inequalities derived from experimental data as physical constraints. Tao et al. [18] proposed a novel turbine blade tip design methodology based on free-form deformation technology, developing a thermo-aerodynamic optimization framework that employs large-scale variable optimization.

The APO algorithm, a novel swarm intelligence algorithm introduced by Wang et al. in 2024 [9], mimics the survival and predation behaviors of the Arctic puffin. While its unique multi-stage foraging strategy presents a promising framework for optimization, empirical studies reveal that the APO, in its original form, is not exempt from the common pitfalls of swarm intelligence algorithms. These include premature convergence, susceptibility to local optima, and an inadequate balance between exploration and exploitation in certain issues, which ultimately limit its convergence precision and practical applicability. To alleviate these issues, several improvements have been proposed. Fakhouri et al. [19] introduced an adaptive differential evolution strategy that utilizes adaptive parameter control and external archives to strengthen its global exploration and convergence efficiency. Sun and Wang [20] proposed a dual-strategy approach combining elite opposition-based learning with adaptive T-distribution mutation to enhance initial population quality and achieve superior balance between global exploration and local exploitation. Zhang [21] introduced an elite reverse learning strategy that significantly improved convergence speed and optimized the effective performance of communication systems. Su and Jiang [22] integrated a Gaussian mutation strategy to balance global exploration with local exploitation. By employing elite opposition-based learning to expand the search space and enhance population diversity. Nevertheless, as stated by the no-free-lunch theorem [23], no single optimization algorithm can dominate all others on every possible problem. Although the aforementioned strategies enhance the optimization performance of APO, it remains prone to local optima and an imbalance between exploration and exploitation.

Therefore, this paper proposes the following improvements to APO: first, a mirror opposition-based learning mechanism with an adaptive mirroring factor is introduced. During the search process, both the current solutions and their dynamically generated mirror opposites are considered as feasible solutions, thus enhancing population diversity and the efficiency of locating optimal solutions, which enhances convergence accuracy. Second, a dynamic differential evolution strategy with an adaptive scaling factor is incorporated to aid in precise optimization and strengthen the algorithm’s ability to escape local optima.

The remainder of this paper is structured as follows: Section 2 presents the fundamental concepts of the standard APO. Section 3 describes the fundamental concepts of the improved APO (IAPO) using mathematical formulations and flowcharts, along with corresponding pseudocode. Section 4 conducts ablation experiments on the proposed improvement strategies and analyzes the performance of the IAPO against eight comparison algorithms. Section 5 applies nine algorithms to three engineering optimization design problems. Finally, the paper concludes and discusses potential directions for future research.

The key contributions of this study are listed below:

(a) A mirror opposition-based learning mechanism is introduced to enhance the quality of the initial population and expand the search scope. An adaptive parameter-driven dynamic differential evolution strategy is also integrated. By adjusting parameters adaptively, the algorithm prioritizes global exploration in early iterations and shifts focus to local exploitation in later stages, thereby achieving an automatic balance between exploration and exploitation and improving the ability to escape local optima.
(b) Comprehensive experiments are conducted on 20 benchmark functions, the CEC 2019 test set, and the CEC 2022 test set, which collectively validate the superiority of the proposed IAPO. Furthermore, simulation experiments on three engineering application problems demonstrate that IAPO outperforms other comparative algorithms in both robustness and practical applicability.

## 2. APO

The APO algorithm comprises three stages: population initialization, exploration (aerial flight), and exploitation (underwater foraging). A behavior transition coefficient regulates the shift between the latter two phases. The corresponding mathematical models are detailed below.

### 2.1. Initialization

As with other metaheuristic algorithms, the process begins with random initialization:

(1) $$X_{i}^{t}=rand\times (ub-lb)+lb,\;i=1,2,\ldots ,N,$$

where for iteration t: $X_{i}^{t}$ denotes the position of the $i^{th}$ Arctic puffin; rand is a random variable uniformly distributed in [0, 1]; and ub and lb respectively define the search space’s upper and lower bounds.

### 2.2. Aerial Flight Phase (Exploration)

Arctic puffins utilize distinctive flight and foraging strategies to cope with demanding environments, the first of which is aerial search. They flexibly switch between aerial and underwater modes to meet nutritional needs and respond to diverse conditions. This section elaborates on two aerial strategies.

The first of which is aerial search, formulated as:

(2) $$\begin{array}{l} Y_{i}^{t+1}=X_{i}^{t}+(X_{i}^{t}-X_{r}^{t})\times L(D)+R\\ R=rand(0.5\times (0.05+rand))\times \alpha \;,\\ s.t.\alpha \sim Normal(0,1) \end{array}$$

where r is a random integer in the range [1, N−1] that excludes i; $X_{i}^{t}$ denotes the $i^{th}$ candidate solution; $X_{r}^{t}$ represents a candidate solution, chosen at random with $X_{i}^{t}\neq X_{r}^{t}$ from the population; L(D) is a Levy flight-generated random number; D corresponds to a specific dimension; and α is a value sampled from a standard normal distribution.

The second strategy involves swooping predation:

(3) $$\begin{array}{l} Z_{i}^{t+1}=Y_{i}^{t+1}\times S\\ S=\tan((rand-0.5)\times \pi ) \end{array}.$$

### 2.3. Underwater Foraging Phase (Exploitation)

The exploitation phase in APO is modeled by the underwater foraging behavior of Arctic puffins, characterized by three strategies: gathering foraging, intensifying search, and predator avoidance.

(1) Gathering foraging

This strategy mimics the collaborative hunting of puffins, which target fish schools near the surface. This cooperative approach boosts efficiency, as individuals can identify rich diving spots and food sources by observing the group. The update formula is:

(4) $$\begin{array}{l} W_{i}^{t+1}=\\ \;\;\left\{\begin{array}{l} X_{r1}^{t}+G\times L(D)\times (X_{r2}^{t}-X_{r3}^{t}),\;rand\geq 0.5\\ X_{r1}^{t}+G\times (X_{r2}^{t}-X_{r3}^{t}),\;rand<0.5 \end{array}\right. \end{array},$$

where G is a cooperation factor, used to regulate the Arctic puffin’s foraging pattern, with G=0.5 in this paper; r1, r2 and r3 are both distinct random integers within [1, N] (excluding i), while $X_{r1}^{t}$, $X_{r2}^{t}$, and $X_{r3}^{t}$ are all candidate solutions randomly selected from the current population.

(2) Intensifying the search

As available food depletes, arctic puffins navigate to new underwater areas to find prey and maintain their nutrient intake. This movement is modeled by:

(5) $$\begin{array}{l} Y_{i}^{t+1}=W_{i}^{t+1}\times (1+f),\\ f=0.1\times (rand-1)\times \frac{\left(T-t\right)}{T}, \end{array}$$

where T is maximum iteration count, t is current iteration, and rand injects randomness into the adaptive factor f. This factor, which is central to the intensifying search phase along with the parameter (1+f), is designed to fine-tune the Arctic puffin’s underwater position. Inspired by the puffin’s foraging flexibility, f dynamically adjusts as iterations progress, enabling the puffin to refine its position based on search status and randomness, thereby enhancing its ability to locate richer food sources.

(3) Avoiding predators

This strategy [9] lies in simulating the collective warning behavior of Arctic puffins. Once a puffin detects a predator, it issues an alarm call, prompting other members to immediately evacuate the danger zone upon receiving the signal. The corresponding mathematical formulation is as follows:

(6) $$\begin{array}{l} Z_{i}^{t+1}=\\ \;\;\left\{\begin{array}{l} X_{i}^{t}+G\times L(D)\times (X_{r1}^{t}-X_{r2}^{t}),\;rand\geq 0.5,\\ X_{i}^{t}+\beta \times (X_{r1}^{t}-X_{r2}^{t}),\;rand<0.5 \end{array}\right. \end{array}$$

where G is a cooperation factor, used to regulate the Arctic puffin’s foraging pattern, with G=0.5 in this paper; β is a random variable drawn from a uniform distribution over [0, 1]; r1 and r2 are both distinct random integers within [1, N] (excluding i), and $X_{r1}^{t}$ and $X_{r2}^{t}$ represent two candidate solutions obtained via random selection from the current population. This approach employs a balancing mechanism that activates two different escape tactics based on the perceived threat level. When  rand≥0.5—simulating the presence of a nearby predator—puffins perform a swift escape maneuver, leading to a significant relocation to avoid danger. This response improves the algorithm’s capacity to escape local optima. On the other hand, when  rand<0.5, puffins switch to a more measured evasion tactic, permitting finer local search. Such a dual-response mechanism enables the algorithm to adapt to various search situations, enabling a balance between exploration and exploitation.

### 2.4. Behavioral Transition Coefficient B

Governed by the behavioral transition coefficient B, the APO algorithm smoothly shifts from global exploration (aerial flight) in early iterations to local exploitation (underwater foraging) in later stages. This dynamic transition ensures a search balance. The coefficient B is defined as:

(7) B=2×log(1/rand)×(1−t/T),

where t is current iteration and T is maximum iteration count. A threshold parameter D=0.5 is used to determine the current search phase: when B>D, the algorithm performs exploration; when B≤D, it switches to exploitation. This dynamic adjustment enables adaptive control over the search process throughout the optimization run.

## 3. IAPO

To address three key limitations of the APO algorithm: sluggish early-stage convergence, a propensity to become trapped in local optima, and an inadequate balance between exploration and exploitation, we propose an IAPO. This enhanced algorithm incorporates a mirror opposition-based learning mechanism and a dynamic differential evolution strategy.

### 3.1. Mirror Opposition-Based Learning Mechanism

In many optimization scenarios, the search process often starts with random initial values and gradually approaches the optimal solution. When initial random values are close to the optimum, the problem can be solved efficiently. However, in the worst case, if the initial values lie opposite to the optimal region, the optimization process becomes time-consuming.

Without prior knowledge, it is difficult to ensure favorable initial solutions. From a logical perspective, the solution space can be explored more effectively by considering both current candidate solutions and their opposites. Introducing opposite solutions as feasible candidates can enhance the efficiency of locating the global optimum. This idea is rooted in the concept of opposition-based learning [24,25] and can be mathematically formulated as follows:

(8) $$R_{i}^{t+1}=(X_{\max}+X_{\min})-X_{i}^{t}.$$

Based on this concept, Yao et al. [26] proposed a mirror opposition-based learning mechanism inspired by convex lens imaging. Unlike conventional opposition-based learning, which produces a fixed opposite solution, the mirror opposition-based mechanism introduces adaptive perturbation via a mirror factor q, enabling dynamic adjustment of opposite solutions. This not only improves optimization accuracy but also maintains convergence speed [27]. The updated position of an individual is given by:

(9) $$M_{i}^{t+1}=\frac{X_{\max}+X_{\min}}{2}+\frac{X_{\max}+X_{\min}}{2q}-\frac{X_{i}^{t}}{q},$$

where q is the mirror factor, $X_{\max}$ is the upper bound of the position, and $X_{\min}$ is the lower bound of the position.

The adaptive update formula for the mirror factor q is:

(10) $$q=10\times (1-2\times {\left(\frac{t}{T}\right)}^{2}).$$

Generally, as a machine learning strategy, opposition-based learning offers the potential to extend existing learning algorithms.

### 3.2. Dynamic Differential Evolution Strategy

The Differential Evolution (DE) algorithm [28] shares its conceptual foundation with genetic algorithms. Both employ random initialization to generate a starting population, utilize fitness values to guide selection, and proceed iteratively through mutation, crossover, and selection operations. However, the Arctic Puffin Optimization (APO) algorithm tends to converge near local optima in the later stages of optimization, increasing the risk of premature convergence. To mitigate this limitation, a dynamic differential evolution strategy is incorporated, which introduces an adaptive scaling factor. The specific procedure is outlined below.

The initial scaling factor E0 and crossover probability CR were determined through preliminary parameter sensitivity experiments within the range [0, 1]. A parameter set demonstrating consistent effectiveness across most test problems was selected to maintain both effective adaptation and population diversity:

(11) E0=0.4,

(12) CR=0.1,

This paper employs the DE/rand/1 mutation strategy, which is selected for its independence from the current best solution. This characteristic helps maintain population diversity and enhances the algorithm’s ability to escape local optima. Furthermore, its simple structure allows smooth integration with the proposed adaptive parameter mechanism.

(13) $$V_{i}^{t+1}=X_{r1}^{t}+E\times (X_{r2}^{t}-X_{r3}^{t}),$$

where r1, r2, r3 are distinct integers randomly selected from the range [1, N] (excluding i), and E is an adaptive scaling factor calculated as follows:

(14) $$E=E0\times 2^{\lambda },$$

(15) $$\lambda =e^{\left(1-T/(T+1-t)\right)}.$$

during the early iterations, the scaling factor E remains relatively high to promote global exploration. As the search progresses, the value E gradually decreases to shift the focus toward local refinement.

Next, the crossover operation is applied to combine the mutation vector $V_{i}^{t+1}$ with the target vector $X_{i}^{t}$ according to certain rules, producing a trial vector:

(16) $$U_{i}^{t+1}=[u_{i,1}^{t+1},\cdots ,u_{i,j}^{t+1},\cdots ,u_{i,D}^{t+1}],$$

where each component is defined by:

(17) $$u_{i,j}^{t+1}=\left\{\begin{array}{l} V_{i,j}^{t+1}\;,\;if\;r_{j}[0,1)\leq CR\;or\;j=r(i)\\ X_{i,j}^{t}\;,\;otherwise \end{array}\right.,$$

where $V_{i,j}^{t+1}$ is the $j^{th}$ dimension value of the $i^{th}$ new parameter individual; $r_{j}[0,1)$ denotes the random number calculated in the $j^{th}$ dimension, and CR is the crossover probability. D denotes the dimension of the population, and r(i) is a random integer in [1, D], thus ensuring the mutation vector contributes at least one component.

Finally, a greedy selection operation is performed based on fitness comparison:

(18) $$X_{i}^{t+1}=\left\{\begin{array}{l} U_{i}^{t+1},\;if\;F(U_{i}^{t+1})<F(X_{i}^{t})\\ X_{i}^{t}\;,\;if\;F(U_{i}^{t+1})\geq F(X_{i}^{t})\; \end{array}\right.,$$

where F(•) denotes the fitness function. This ensures that the population evolves toward improved solutions over successive generations, maintaining selection pressure toward higher-quality regions of the search space.

### 3.3. Algorithm Flow

The pseudocode of IAPO is shown as Algorithm 1:
**Algorithm 1. IAPO****Input:** Population size *N*, maximum iterations *T*, problem dimension *D*.**Output:** Global best position *X_gbest_*, global best fitness *f*(*X_gbest_*).1.   Initialize parameters *N*, *T*; randomly initialize population $X_{i}^{0}$, evaluate fitness*f*($X_{i}^{0}$) for each individual.2.   Initialize the local best solution *f*(*X_lbest_*) and position *X_lbest_*.3.   Initialize the global best solution *f*(*X_gbest_*) and position *X_gbest_*.4.     **for**
*t* = 1 → *T*5.           Calculate the mirror factor *q* using Formula (10).6.           **for**
*i* = 1 → *N*7.                 Calculate the new position $M_{i}^{t+1}$ using Formula (9).8.                 **if**
*f*($M_{i}^{t+1}$) < *f*($X_{i}^{t}$)9.                      $X_{i}^{t+1}$ = $M_{i}^{t+1}$, *f*($X_{i}^{t+1}$) = *f*($M_{i}^{t+1}$)10.               **end if**11.         **end for**12.         **for**
*i* = 1 → *N*13.         Calculate the behavior transition coefficient *B* using Formula (7).14.         **if**
*B* > 0.515.            Calculate the new positions $Y_{i}^{t+1}$ and $Z_{i}^{t+1}$ using formulas (2)–(3)16.            Update the individual position through comparison 17.         **else if**18.            Calculate the new positions $W_{i}^{t+1}$, $Y_{i}^{t+1}$, and $Z_{i}^{t+1}$ using formulas19.            (4)–(6); Update the individual position through comparison20.         **end if**21.    **end for**22.    Update *f*(*X_lbest_*), *X_lbest_*, *f*(*X_gbest_*), and *X_gbest_*.23.    **for**
*i* = 1 → *N*24.         Calculate the mutation vector $V_{i}^{t+1}$ using formulas (13)–(15).25.         Calculate the trial vector $U_{i}^{t+1}$ using formulas (16)–(17). 26.    **end for**27.    **for**
*i* = 1 → *N*28.         **if**
*f*($U_{i}^{t+1}$) < *f*($X_{i}^{t}$)29.            $X_{i}^{t+1}$ = $U_{i}^{t+1}$, *f*($X_{i}^{t+1}$) = *f*($U_{i}^{t+1}$)30.      **end if**31.    **end for**32.    Update *f*(*X_gbest_*) and *X_gbest_*.33.  **end for**34.  **Return:**
*X_gbest_* and *f*(*X_gbest_*) *f*(*X_gbest_*)

**Figure 1:**
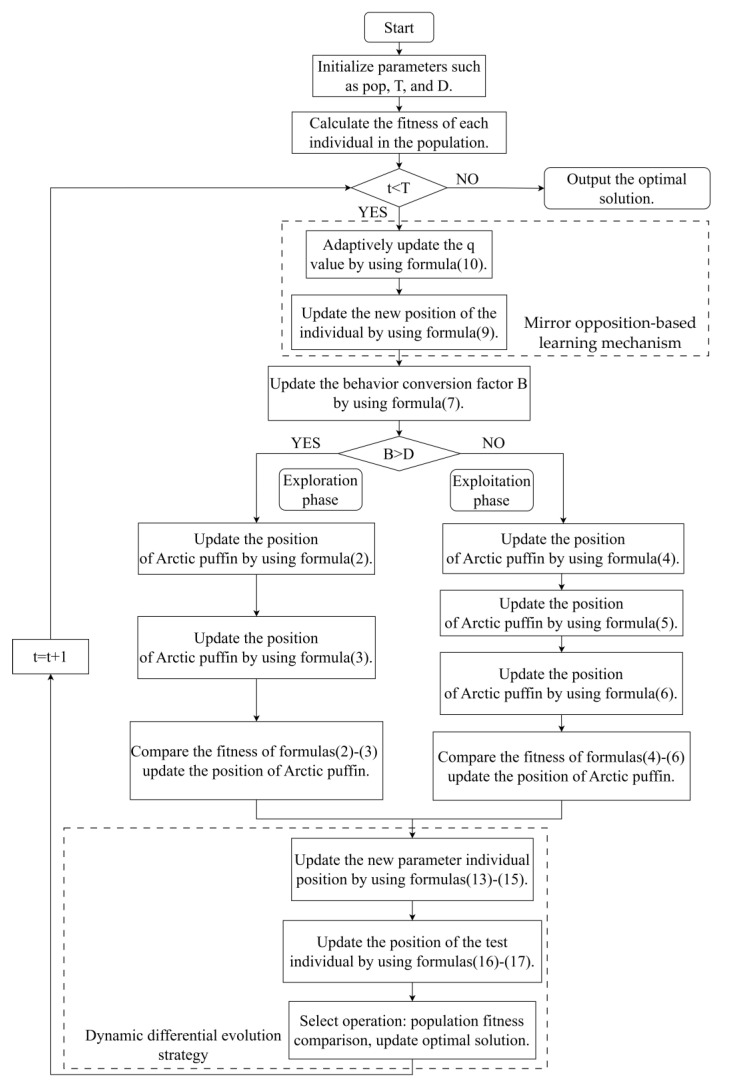
Flowchart of the overall solution process of the IAPO algorithm.

To clearly describe the overall solution logic of IAPO, a flowchart of this algorithm is drawn as Figure 1.

### 3.4. Time Complexity Analysis

Time complexity serves as a key metric for evaluating algorithmic performance, as it governs code execution efficiency. The following analysis compares the time complexity between the standard APO and the improved IAPO algorithm.

Assuming the population size of Arctic puffins is pop, the maximum number of iterations is T, and the dimension is D.

(1) Time complexity of APO

The APO algorithm operates through three sequential phases: population initialization, aerial flight for global exploration, and underwater foraging for local exploitation. The time complexities of these three stages are as follows:

Initialization phase: Iterate N times, generating a D-dimensional vector each time, with a time complexity of $O\left(N\cdot D\right)$.

Exploration phase: Includes two strategies—aerial search and swooping predation—each looping N times, generating a new *D*-dimensional solution each time, with a complexity of $O\left(N\cdot D\right)$. After T iterations, the time complexity is $O\left(N\cdot D\cdot T\right)$.

Exploitation Phase: This phase is characterized by the application of three distinct strategies: gathering foraging, intensifying search, and predator avoidance, with position updates for all three strategies requiring pop×D calculations each time. After T iterations, the time complexity is $O\left(N\cdot D\cdot T\right)$.

Hence, the combined time complexity of all algorithmic components amounts to $O\left(N\cdot D\right)+O\left(N\cdot D\cdot T\right)+O\left(N\cdot D\cdot T\right)\approx O\left(N\cdot D\cdot T\right)$.

(2) Complexity analysis of the mirror opposition-based learning mechanism

This approach diversifies the search space by creating opposition-based counterparts for the population, with computations involving inverse calculations for each dimension of every individual in the population, single execution has a complexity of $O\left(N\cdot D\right)$. After T iterations, this results in a total time complexity of $O\left(N\cdot D\cdot T\right)$.

(3) Time complexity of the dynamic differential evolution strategy

The core operations of the dynamic differential evolution strategy include mutation, crossover, and selection. The time complexities of these three operations during the iteration phase are as follows:

Mutation operation: For each individual, select 3 random individuals to calculate a D-dimensional differential vector, with a complexity of $O\left(N\cdot D\right)$.

Crossover operation: Perform crossover operations on each dimension of each individual according to probability, with a complexity of $O\left(N\cdot D\right)$.

Selection operation: Compare the fitness of the target individual with that of the trial individual, with a complexity of $O\left(N\cdot D\right)$.

Total iteration complexity: After T iterations of the above three operations, the total time complexity is $O\left(N\cdot D\cdot T\right)\times 3\approx O\left(N\cdot D\cdot T\right)$.

(4) Time complexity of IAPO

The total time complexity of the IAPO algorithm is the sum of the time complexities of the APO algorithm, the mirror-based learning mechanism, and the dynamic differential evolution strategy: $O\left(N\cdot D\cdot T\right)+O\left(N\cdot D\cdot T\right)+O\left(N\cdot D\cdot T\right)\approx O\left(N\cdot D\cdot T\right).$ In summary, it is found that the time complexity of IAPO is of the same order as that of standard APO and does not significantly increase the computational burden of the algorithm.

## 4. Simulation Experiments and Result Analysis

The performance of the proposed IAPO was rigorously evaluated using a comprehensive set of test functions, comprising 20 classical benchmarks, the CEC 2019 set, and the CEC 2022 set. The basic information of these test sets is provided in Table 1, Table 2 and Table 3, respectively.

The 20 benchmark functions listed in Table 1 [29] are divided into three groups with distinct characteristics to comprehensively assess the algorithm’s performance: F1–F7 are unimodal functions, which are characterized by the presence of a single global optimum and the absence of local optima. These functions primarily test the convergence speed of the algorithm in straightforward optimization scenarios. F8–F13 [30] are multimodal functions, which contain one global optimum and multiple local optima. They are used to evaluate the algorithm’s ability to balance global exploration and local exploitation. F14–F20 [31] are fixed-dimensional multimodal functions, which test the robustness of algorithms in exploration and exploitation capabilities under constrained dimensions. The CEC 2019 test set [32], shown in Table 2, includes 10 objective functions. Functions F1 to F3 vary in dimension and search range, while F4 to F10 are 10-dimensional. The theoretical optimum for each function in this set is 1. The CEC 2022 test set [33], detailed in Table 3, comprises twelve functions with dimensions of 10 and 20, encompassing unimodal (F1), basic (F2–F5), hybrid (F6–F8), and composition (F9–F12) types. Many of the CEC 2019 and CEC 2022 functions are multimodal [34] and present significant optimization challenges.

To ensure fairness in comparisons, all algorithms were coded in MATLAB 2022a and run on a computer with the following configuration: an AMD Ryzen 5 processor and 16 GB of RAM. The parameters were consistent across all algorithms: population size N = 30 and maximum iterations T = 500. Each algorithm was independently run 30 times. The best value (Best), mean value (Mean), and standard deviation (Std) of the results were used as evaluation indicators.

### 4.1. Ablation Study on Improvement Strategies

IAPO enhances the standard APO by incorporating two strategies: a mirror opposition-based learning mechanism and a dynamic differential evolution strategy. To analyze the contribution of each strategy, two variant algorithms were constructed:

(1) BAPO: APO enhanced with the mirror opposition-based learning mechanism.

(2) DAPO: APO enhanced with the dynamic differential evolution strategy.

**Figure 2:**
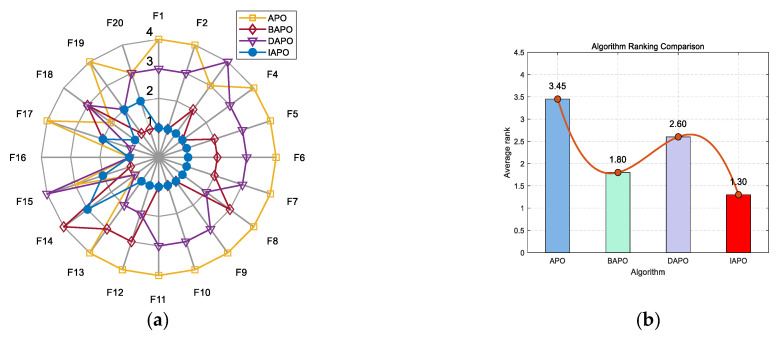
Comparison of ablation study results on the 20 benchmark functions. (**a**) Radar chart illustrates the ranking of each algorithm across all test functions (in the radar charts, each axis represents a test function, with larger values indicating poorer performance. A smaller enclosed area suggests better overall performance). (**b**) Bar chart compares the overall average rankings of the four algorithms on the test set.

**Figure 3:**
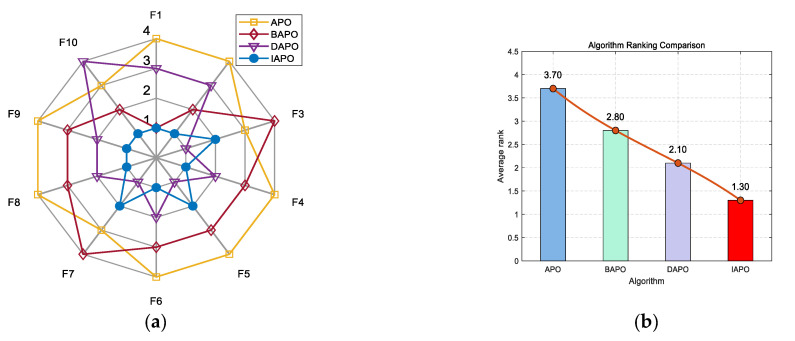
Comparison of ablation study results on the CEC 2019 test set. (**a**) Radar chart illustrates the ranking of each algorithm across all test functions. (**b**) Bar chart compares the overall average rankings of the four algorithms on the test set.

**Figure 4:**
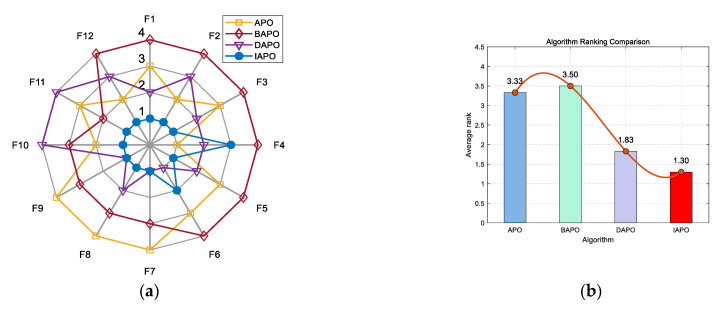
Comparison of ablation study results on the 10-dimensional CEC 2022 test set. (**a**) Radar chart illustrates the ranking of each algorithm across all test functions. (**b**) Bar chart compares the overall average rankings of the four algorithms on the test set.

**Figure 5:**
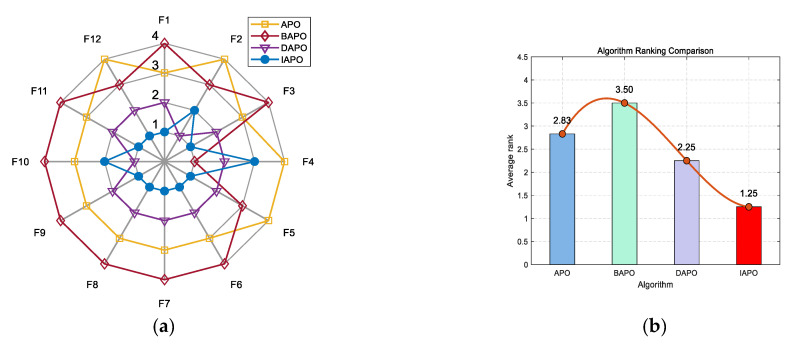
Comparison of ablation study results on the 20-dimensional CEC 2022 test set. (**a**) Radar chart illustrates the ranking of each algorithm across all test functions. (**b**) Bar chart compares the overall average rankings of the four algorithms on the test set.

IAPO, APO, BAPO, and DAPO were evaluated on three distinct sets: 20 benchmark functions, along with the CEC 2019 and CEC 2022 test sets. The results of the ablation study are visualized using radar charts and ranking bar charts: Figure 2 corresponds to the 20 benchmark functions, Figure 3 to CEC 2019, and Figure 4 and Figure 5 to the 10- and 20-dimensional CEC 2022 sets, respectively.

In the accompanying radar charts, each radial axis corresponds to a specific test function, with larger values indicating poorer performance. A smaller enclosed area suggests better overall performance. Across all figures, IAPO (blue circle) consistently occupies positions closer to the center and covers the smallest area, indicating superior performance and stability. Similarly, in the ranking bar charts, IAPO consistently ranks first, validating the effectiveness of integrating both improvement strategies.

### 4.2. Experiments on Benchmark Functions

For a comprehensive evaluation of IAPO’s performance, comparative experiments employed the suite of benchmark functions detailed in Table 1. The following representative swarm intelligence algorithms were selected for comparison: the well-established Whale Optimization Algorithm (WOA) [35], Harris Hawks Optimization (HHO) [36], and Particle Swarm Optimization (PSO) [8]; more recently proposed algorithms, including the Water Uptake and Transport in Plants (WUTP) [37] algorithm and the Rüppell’s Fox Optimizer (RFO) [38]; two improved algorithms for APO the JAPO algorithm [19] and the ETAAPO algorithm [20], and the standard Arctic Puffin Optimization (APO) algorithm. Parameter settings for each algorithm were set as follows: IAPO(E0=0.4,  CR=0.1, G=0.5, D=0.5), WOA(a=2→0, b=1), PSO(w=0.7, c1=1.5, c2=2.0), WUTP(p=0.5, β=0.1), RFO(β=0.1, L=100), APO(F=0.5, D=0.5), JAPO(uF=0.5, uCR=0.5, p0=0.05, F=0.5, B=0.5), ETAAPO(F=0.5, B=0.5). The optimization results of all algorithms on 30-dimensional functions F1–F13 and fixed-dimensional functions F14–F20 are summarized in Table 4. The bold numbers in the table indicate the optimal values.

For the unimodal functions F1–F4, IAPO achieved the theoretical optimum value of zero for the best, mean, and standard deviation of the results, significantly outperforming the other eight algorithms. This demonstrates IAPO’s strong global search capability and stability. On functions F5–F7, IAPO also ranked first in all three evaluation metrics. On the multimodal functions F8–F13, which contain numerous local optima, IAPO attained the theoretical optimum on F8 with the smallest standard deviation, indicating a high ability to avoid local optima. On F9–F11, both HHO and IAPO delivered excellent and identical results. For F12 and F13, IAPO again outperformed all other algorithms across all metrics.

Across the subset of fixed-dimensional multimodal functions (F14–F20), the performance of the nine algorithms was comparable on F16. IAPO performed relatively poorly on F14, ranking sixth. On F18 and F19, IAPO’s standard deviation was slightly higher than that of ETAAPO and JAPO, placing it second. However, on F15, F17, and F20, IAPO achieved the best convergence accuracy, the highest average performance, and the smallest standard deviation, thus validating its robustness and effective balance between exploration and exploitation for solving fundamental problems.

**Figure 6:**
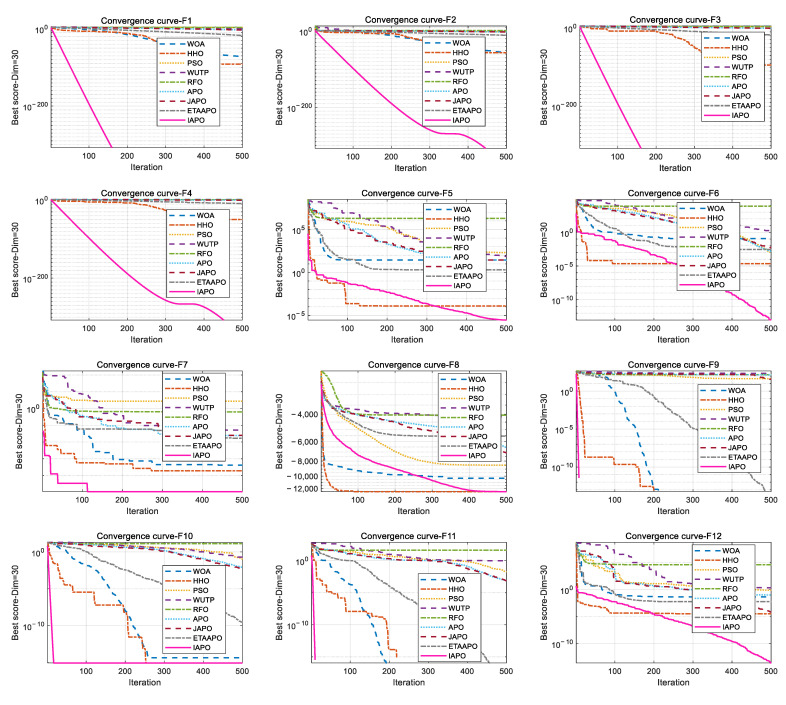

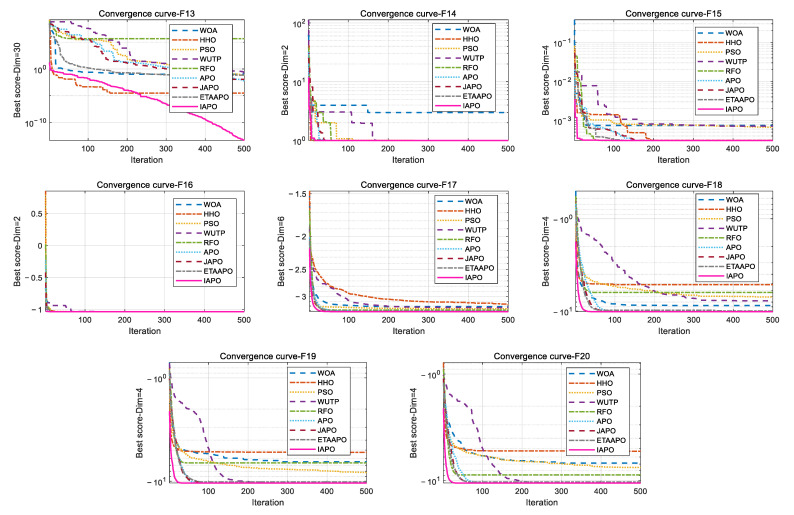
Comparative convergence behavior of the nine algorithms on 20 benchmark functions.

To further illustrate the convergence behavior, the convergence curves of IAPO and the other algorithms are plotted in Figure 6.

Figure 6 presents a comparative view of the convergence characteristics, highlighting IAPO’s performance against other algorithms. For most test functions—including F1 to F4, F7, F9 to F11, F15, and F17 to F20—the IAPO curve converges rapidly and attains high precision early in the process. By the 50th iteration, IAPO already shows a clear advantage in both convergence speed and convergence accuracy. On functions F5, F6, F12, and F13, IAPO reaches the optimal value after approximately 200 iterations, and its curve continues to decline gradually even up to 500 iterations, reflecting sustained search refinement. Notably, on F7 and F8, IAPO repeatedly escapes local optima and finds better solutions, demonstrating a strong ability to avoid premature convergence. Overall, these results confirm that IAPO maintains high optimization accuracy and faster convergence speed across a variety of benchmark problems.

**Figure 7:**
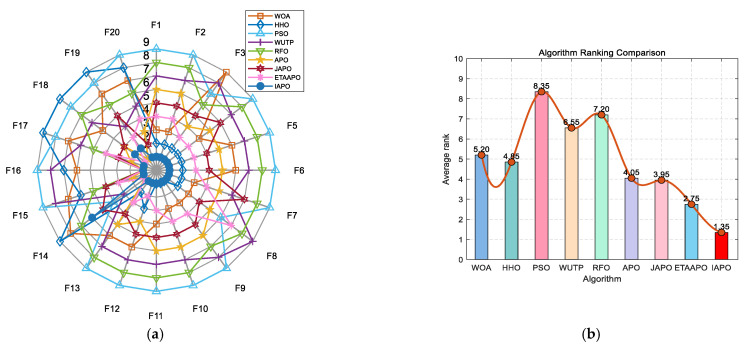
Performance ranking of IAPO and comparison algorithms on the 20 benchmark test functions. (**a**) Radar chart illustrates the ranking of each algorithm across all test functions. (**b**) Bar chart compares the overall average rankings of the four algorithms on the test set.

Figure 7 presents a radar chart and a bar chart summarizing the ranking of each algorithm across the 20 benchmark functions. In the radar chart, IAPO (blue circles) encloses the smallest area and is consistently positioned closer to the center—indicating superior and more stable performance. Similarly, the bar chart confirms that IAPO achieves the highest overall ranking, underscoring its effectiveness compared to the other algorithms.

### 4.3. Experiments on CEC 2019 Test Functions

Following the experimental setup used for the benchmark functions in Section 4.2, the proposed IAPO algorithm was compared with WOA, HHO, PSO, WUTP, RFO, APO, JAPO, and ETAAPO. The corresponding results of the CEC 2019 test set are detailed in Table 5, with all parameter configurations consistent with those described in Section 4.2.

As shown in Table 5, IAPO achieves competitive results on most test functions. Notably, it generally exhibits smaller standard deviations than the other algorithms, indicating more stable performance. Among the ten CEC 2019 functions, IAPO ranks first on eight functions (F1, F2, F4–F8, and F10). On function F3, IAPO’s best value is slightly lower than that of JAPO, but its mean accuracy is the highest among all algorithms. On F9, IAPO’s mean value ranks third, behind JAPO and ETAAPO. Overall, IAPO demonstrates clear superiority in 80% of the functions, confirming its effectiveness in handling complex optimization problems.

**Figure 8:**
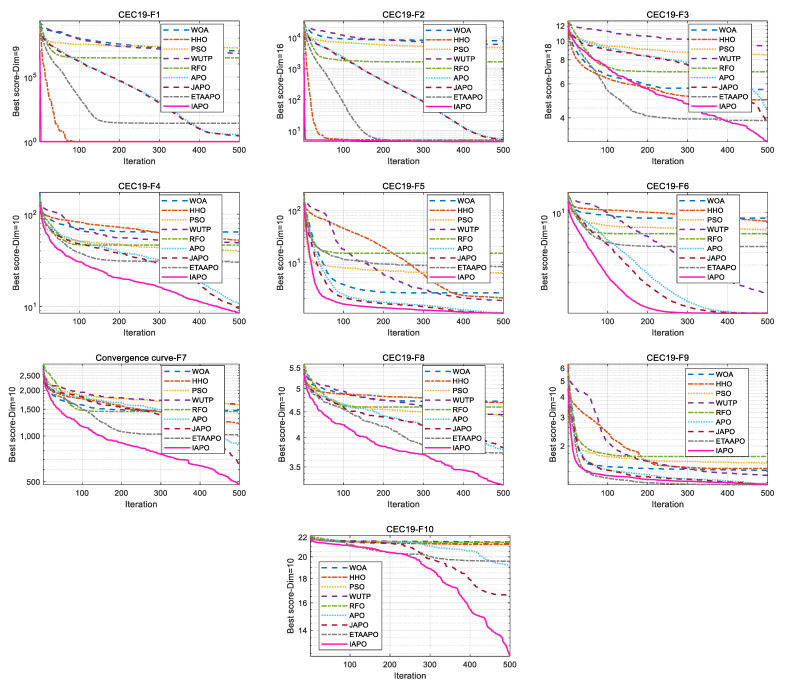
Comparative convergence behavior of the nine algorithms on the CEC 2019 set.

Figure 8 displays the convergence curves of IAPO and the comparison algorithms on the CEC 2019 test set. The results show that IAPO converges rapidly to lower fitness values across most functions. On F1, F2, F5, and F6, it reaches high precision within a small number of iterations. By the 50th iteration, IAPO already shows significantly better accuracy and convergence speed than the other algorithms. On functions such as F3, F4, F8, and F10, IAPO repeatedly escapes local optima and continues to refine the solution, demonstrating strong local avoidance capability.

**Figure 9:**
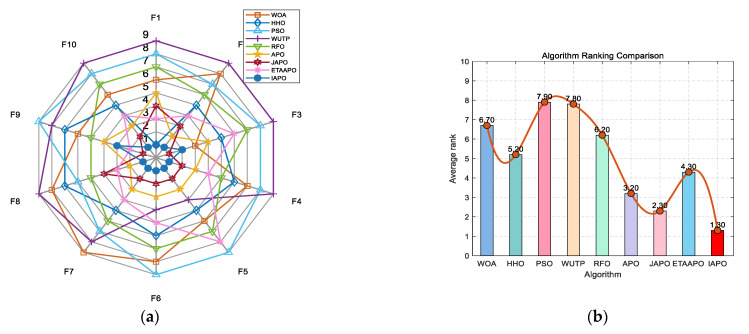
Relative performance ranking of IAPO and competing algorithms on the CEC 2019 test set. (**a**) Radar chart illustrates the ranking of each algorithm across all test functions. (**b**) Bar chart compares the overall average rankings of the four algorithms on the test set.

Figure 9 summarizes the ranking of each algorithm using a radar chart and a bar chart. In the radar chart, IAPO (blue circle) covers the smallest area and is positioned closest to the center, reflecting its superior and consistent performance. The bar chart further confirms that IAPO achieves the highest overall ranking.

### 4.4. Experiments on the CEC 2022 Test Set

The IAPO algorithm was compared with eight other swarm intelligence algorithms—WOA, HHO, PSO, WUTP, RFO, APO, JAPO, and ETAAPO—using the same parameter settings as in Section 4.2. The optimization results for the 10- and 20-dimensional CEC 2022 sets are presented in Table 6 and Table 7, respectively.

As shown in Table 6, for the 10-dimensional case, IAPO achieved the first rank on 10 out of 12 functions. On function F2, IAPO ranked second, slightly behind APO, while on F4, it placed third with a marginal difference from the top two algorithms, APO and JAPO. In the 20-dimensional setting (Table 7), IAPO ranked first on 11 functions, and second on F4, closely following JAPO. Overall, IAPO exhibited superior performance on 87.5% of the functions in the CEC 2022 test set.

**Figure 10:**
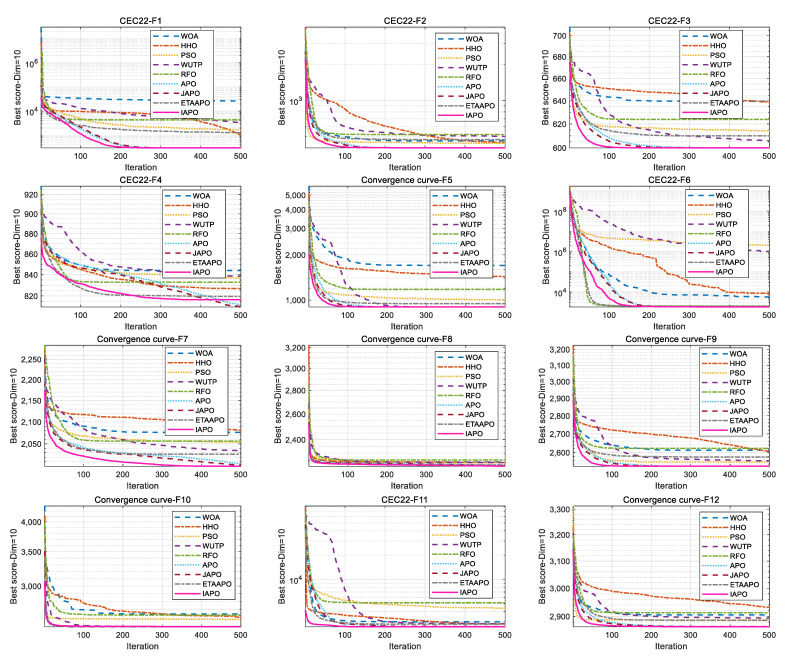
Comparative convergence behavior of the nine algorithms on the 10D CEC 2022 set.

**Figure 11:**
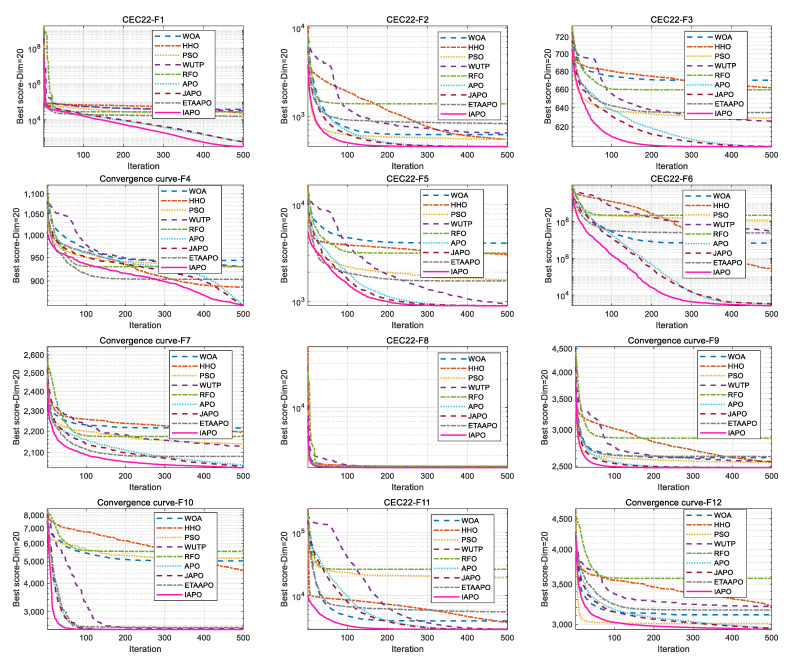
Comparative convergence behavior of the nine algorithms on the 20D CEC 2022 set.

Figure 10 and Figure 11 illustrate the convergence curves of IAPO and the comparison algorithms for the 10- and 20-dimensional sets, respectively. In both figures, IAPO converges more rapidly than the other algorithms, with smoother convergence curves and fewer instances of stagnation in local optima. This indicates that IAPO possesses not only faster convergence but also stronger global search capability and robustness for addressing challenging high-dimensional optimization problems.

**Figure 12:**
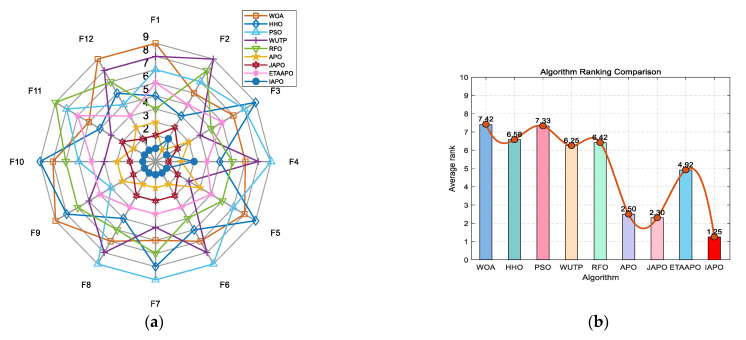
Performance ranking of IAPO and comparison algorithms on the 10-dimensional CEC 2022 test set. (**a**) Radar chart illustrates the ranking of each algorithm across all test functions. (**b**) Bar chart compares the overall average rankings of the four algorithms on the test set.

**Figure 13:**
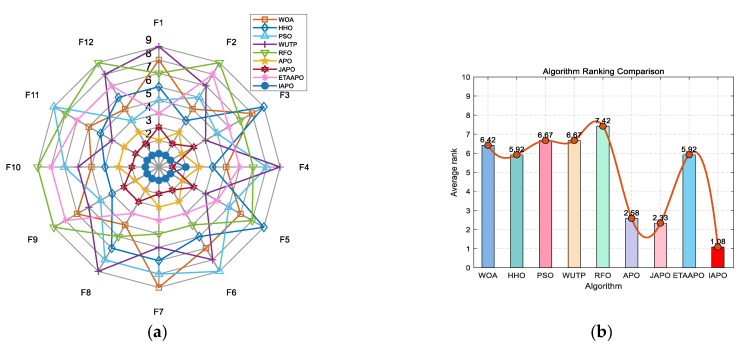
Performance ranking of IAPO and comparison algorithms on the 20-dimensional CEC 2022 test set. (**a**) Radar chart illustrates the ranking of each algorithm across all test functions. (**b**) Bar chart compares the overall average rankings of the four algorithms on the test set.

Figure 12 and Figure 13 present the ranking comparisons based on the 10- and 20-dimensional CEC 2022 sets, respectively. In the radar charts, IAPO (blue circle) occupies the smallest area and is consistently positioned near the center, reflecting its stable and superior performance across most functions. The accompanying bar charts confirm that IAPO achieves the highest overall ranking, further validating its effectiveness.

In summary, IAPO exhibits rapid initial convergence, which is facilitated by the introduced mirror opposition-based learning. This mechanism creates a high-quality, diverse initial population, providing a favorable starting point for the optimization process. Furthermore, IAPO demonstrates exceptional performance on complex multimodal functions. This capability is primarily due to its adaptive differential evolution strategy, which dynamically balances global exploration and local exploitation throughout the iterations. Effectively introducing new individuals enables the algorithm to escape local optima consistently.

### 4.5. Nonparametric Statistical Analysis Using Wilcoxon Rank-Sum and Friedman Tests

To provide a statistically rigorous comparison of algorithm performance beyond basic metrics such as the best, mean, and standard deviation, this experiment employs the Wilcoxon rank-sum test at a 95% confidence level. This test assesses whether the differences between IAPO and each comparison algorithm are statistically significant. The test was implemented in MATLAB 2022a using the ranksum(x,y) function. A *p*-value below the 0.05 threshold indicates a statistically significant difference between the two algorithms, whereas a value above it suggests no such significance. In the results, the symbols “+”, “=“, and “−” denote that IAPO performs significantly better, shows no significant difference, or performs significantly worse than the comparison algorithm, respectively.

The Wilcoxon test statistics for IAPO across the different test sets are summarized in Table 8. The results, presented as the number of functions where IAPO wins/ties/loses against the comparison algorithms, are 142/15/3 for the 20 benchmark functions, 70/8/2 for the CEC 2019 test set, 91/3/2 for the 10-dimensional CEC 2022 set, and 94/2/0 for the 20-dimensional CEC 2022 set. The prevalence of *p*-values less than 0.05 indicates that IAPO’s performance is significantly different from that of the other algorithms across most functions.

To further validate the overall performance ranking of IAPO against the comparison algorithms, the non-parametric Friedman test was conducted. For each test function, the algorithms were ranked based on their optimization results (with Rank 1 assigned to the best performer). If multiple algorithms achieved identical results, they were assigned an average rank. The test was implemented in MATLAB 2022a using the friedman(data) function. The Friedman test compares the average ranks of all algorithms across all functions, where a lower average rank indicates superior overall optimization performance. The calculation of the average rank is shown in Equation (18).

(19) $$\left\{\begin{array}{l} rank_{a}^{j}=\frac{1}{N_{r}}\sum_{i=1}^{N_{r}}R_{i}^{j}\\ Averank_{j}=\frac{1}{N_{tf}}\sum_{a=1}^{N_{tf}}rank_{a}^{j} \end{array}\right.,$$

where $rank_{a}^{j}$ denotes the average rank of the $j^{th}$ algorithm over $N_{r}$ independent runs on the $a^{th}$ test function. $N_{r}$ is the number of independent runs, $R_{i}^{j}$ denotes the rank of the $j^{th}$ algorithm among all algorithms in the $i^{th}$ run, $Averank_{j}$ is the overall average rank of the $j^{th}$ algorithm, and $N_{tf}$ is the number of test functions.

Table 9 shows the Friedman test results comparing IAPO with the eight comparison algorithms. The results show that IAPO achieved the lowest average ranks across all test sets: 1.15 on the 20 benchmark functions, 2.02 on the CEC 2019 test set, 1.50 on the 10-dimensional CEC 2022 set, and 1.48 on the 20-dimensional CEC 2022 set. With an overall rank first among all nine algorithms, IAPO demonstrates statistically superior performance in optimization against its comparison algorithms.

## 5. Experiments in Practical Engineering Optimization Problems

The applicability of IAPO to practical engineering design is further assessed using three constrained optimization problems: the planetary gear train design, the heat exchanger network design (case 1), and the blending-pooling-separation problem. The performance of IAPO is compared with that of WOA, HHO, PSO, WUTP, RFO, APO, JAPO, and ETAAPO.

All experiments were conducted in MATLAB 2022a under identical hardware and software conditions. Employing a population size of 30 and a maximum of 300 iterations, each algorithm underwent 30 independent runs. The Best, Mean, and Standard Deviation (Std) values were recorded as evaluation metrics.

### 5.1. Planetary Gear Train Design Problem

**Figure 14:**
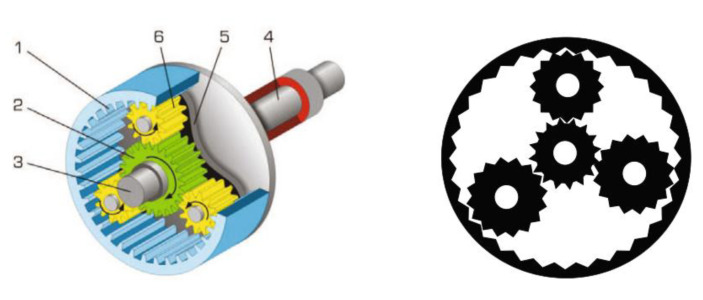
Schematic view of planetary gear train design problem.

The planetary gear train design problem [39] is a constrained optimization task in mechanical power transmission systems, with a schematic shown in Figure 14. Formulated for automotive applications, this problem aims to minimize the maximum gear ratio error in a planetary transmission system. The solution entails determining the total gear tooth count, modeled through six integer decision variables. (number of gear teeth $x_{1}\sim x_{6}=N_{1}\sim N_{6}$) and three discrete variables (gear module $x_{7}$, $x_{8}$ = $m_{1}$, $m_{2}$, number of planetary gears $x_{9}=p$), totaling 9 variables subject to 11 constraints.

Mathematically, the optimization model can be expressed as:

Consider variable $x\;=\;(x_{1},x_{2},x_{3},x_{4},x_{5},x_{6},x_{7},x_{8},x_{9})=(N_{1},N_{2},N_{3},N_{4},N_{5},N_{6},m_{1},m_{2},p)$

Minimize $f(x)=\max|i_{k}-i_{0k}|,\;k=\{1,2,\ldots ,R\}.$

Where $i_{1}=\frac{N_{6}}{N_{4}},\;i_{01}=3.11,\;i_{2}=\frac{N_{6}(N_{1}N_{3}+N_{2}N_{4})}{N_{1}N_{3}(N_{6}+N_{4})},\;I_{R}=\frac{N_{2}N_{6}}{N_{1}N_{3}},\;i_{02}=1.84$

Subject to

$$\begin{array}{l} g_{1}(x)=m_{2}(N_{6}+2.5)-D_{\max}\leq \;0,\\ g_{2}(x)=m_{1}(N_{1}+N_{2})+m_{1}(N_{2}+2)-D_{\max}\leq 0,\\ g_{3}(x)=m_{2}(N_{4}+N_{5})+m_{2}(N_{5}+2)-D_{\max}\leq 0,\\ g_{4}(x)=m_{1}(N_{1}+N_{2})-m_{2}(N_{6}+N_{3})|-m_{1}-m_{2}\leq 0,\\ g_{5}(x)=-(N_{1}+N_{2})\sin(\pi /p)+N_{2}+2+\delta_{22}\leq 0,\\ g_{6}(x)=-(N_{6}-N_{3})\sin(\pi /p)+N_{3}+2+\delta_{33}\leq 0,\\ g_{7}(x)=-(N_{4}+N_{5})\sin(\pi /p)+N_{5}+2+\delta_{55}\leq 0,\\ g_{9}(x)=N_{4}-N_{6}+2N_{5}+2\delta_{56}+4\leq 0,\\ g_{10}(x)=N_{4}-N_{6}+N_{4}+2\delta_{34}+4\leq 0,\\ h_{1}(x)=\frac{N_{6}-N_{4}}{p}=integer. \end{array}$$

where

$$\begin{array}{l} \delta_{22}=\delta_{33}=\delta_{55}=\delta_{35}=\delta_{56}=0.5,\;\beta =\frac{\cos^{-1}{\left(N_{4}+N_{5}\right)}^{2}+{\left(N_{6}-N_{3}\right)}^{2}-{\left(N_{3}+N_{5}\right)}^{2}}{2(N_{6}-N_{3})(N_{4}+N_{5})},\\ D_{\max}=220. \end{array}$$

With bounds

$$\begin{array}{l} p=(3,4,5),\;m_{1},\;m_{2}=(1.75,2.0,2.25,2.5,2.75,3.0),\;17\leq N_{1}\leq 96,\\ 14\leq N_{2}\leq 54,\;14\leq N_{3}\leq 51,\;17\leq N_{4}\leq 46,\;14\leq N_{5}\leq 51,\;48\leq N_{5}\leq 124. \end{array}$$

**Figure 15:**
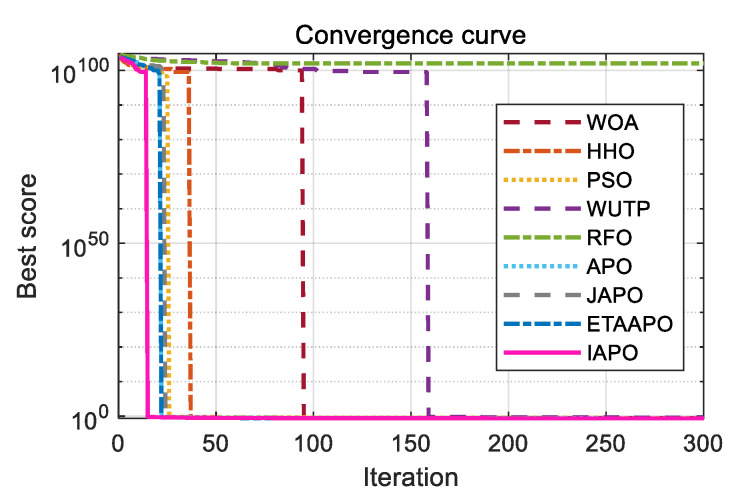
Convergence curves of the nine algorithms applied to the planetary gear train design problem.

The optimization results of IAPO and other comparison algorithms on the planetary gear design problem are summarized in Table 10, including the recorded best, mean, and standard deviation values. The corresponding optimal design variable sets for each algorithm are summarized in Table 11, which indicates that IAPO achieves the smallest values in all three performance metrics. Specifically, IAPO obtains the best objective value of 5.2559 × 10^−1^ with the following optimal variable set: (35, 26, 25, 24, 20, 87, 1.5461, 2.0606, 1.4739). The convergence curve is illustrated in Figure 15, where IAPO exhibits the fastest convergence rate, with its curve consistently occupying the lowest position. These results demonstrate that IAPO is well-suited for this engineering problem and effectively minimizes the maximum gear ratio error.

### 5.2. Heat Exchanger Network Design Problem (Case 1)

The heat exchanger network design problem [40] involves the optimal configuration design of heat exchanger structures, representing a complex engineering optimization challenge. The problem requires heating a cold fluid stream using three hot fluids with distinct inlet temperatures, aiming to minimize the heat transfer area of the exchanger. This represents the first instance of a heat exchange network design problem. In this case, the formulation includes two nonlinear and six linear equality constraints, and a nonlinear objective function. Moreover, nine linear inequality constraints are incorporated to account for temperature limitations.

Mathematically, the optimization model can be expressed as:

Minimize: $f(x)=35x_{1}^{0.6}+35x_{2}^{0.6}$.

Subject to:

$$\begin{array}{l} h_{1}(x)=200x_{1}x_{4}-x_{3}=0,\\ h_{2}(x)=200x_{2}x_{6}-x_{5}=0,\\ h_{3}(x)=x_{3}-10000(x_{7}-100)=0,\\ h_{4}(x)=x_{5}-10000(300-x_{7})=0,\\ h_{5}(x)=x_{3}-10000(600-x_{8})=0,\\ h_{6}(x)=x_{5}-10000(900-x_{9})=0,\\ h_{7}(x)=x_{4}\ln(x_{8}-100)-x_{4}\ln(600-x_{7})-x_{8}+x_{7}+500=0,\\ h_{8}(x)=x_{6}\ln(x_{9}-x_{7})-x_{6}\ln(600)-x_{9}+x_{7}+600=0, \end{array}$$

With bounds

$$\begin{array}{l} 0\leq x_{1}\leq 10,\;0\leq x_{2}\leq 200,\;0\leq x_{3}\leq 100,\;0\leq x_{4}\leq 200,\;\\ 1000\leq x_{5}\leq 2000000,\;0\leq x_{6}\leq 600,\;100\leq x_{7}\leq 600,\;100\leq x_{8}\leq 600,\;\\ 100\leq x_{9}\leq 900. \end{array}$$

**Figure 16:**
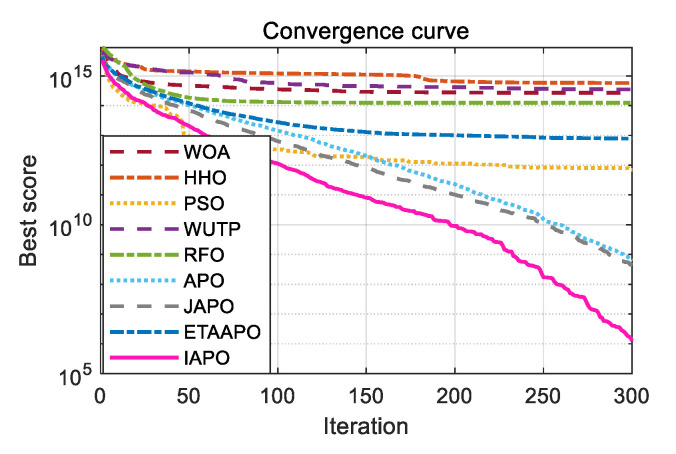
Convergence curves of the nine algorithms applied to the heat exchanger network design problem (case 1).

The optimization results of the IAPO and comparison algorithms on the heat exchanger network design problem (Case 1) are summarized in Table 12 and Table 13. Table 12 presents the best, mean, and standard deviation values, while Table 13 lists the minimum area and corresponding optimal variables obtained by each algorithm. The results demonstrate that IAPO achieves the smallest values in all three performance metrics, attaining the minimum heat transfer area of 1.09E+03 with the optimal variable set: (0.809, 98.130, 78.244, 0.483, 1,999,921.277, 101.902, 100.008, 599.992, 700.008). The convergence curves, illustrated in Figure 16, where IAPO exhibits the fastest convergence rate, with its curve consistently occupying the lowest position. These results confirm that IAPO is well-suited for this problem and effectively minimizes the heat exchanger’s heat transfer area.

### 5.3. Blending-Pooling-Separation Problem

This problem [16] describes a typical chemical engineering unit operation that separates a three-component feed mixture into two multi-component product streams through a network of separators and splitting/blending/pooling, ultimately yielding high-purity products. The operating cost of each separator is proportional to its flow rate, and the process must satisfy mass balance constraints around each unit operation. The objective is to minimize total cost while adhering to material balances, component allocations, and flow constraints. The problem includes 15 nonlinear equality constraints, 17 linear equality constraints, five linear inequality constraints, and a linear objective function. Variables x1 to x38 denote flow indicators.

Mathematically, the optimization model can be expressed as:

Minimize: $f(x)=0.9979+0.00432x_{5}+0.01517x_{13}$.

Subject to:

$$\begin{array}{l} h_{1}(x)=x_{4}+x_{3}+x_{2}+x_{1}=300,\\ h_{2}(x)=x_{6}-x_{8}-x_{7}=0,\\ h_{3}(x)=x_{9}-x_{11}-x_{10}-x_{12}=0,\\ h_{4}(x)=x_{14}-x_{16}-x_{17}-x_{15}=0,\\ h_{5}(x)=x_{18}-x_{20}-x_{19}=0,\\ h_{6}(x)=x_{5}x_{21}-x_{6}x_{22}-x_{9}x_{23}=0,\\ h_{7}(x)=x_{5}x_{24}-x_{6}x_{25}-x_{9}x_{26}=0,\\ h_{8}(x)=x_{5}x_{27}-x_{6}x_{28}-x_{9}x_{29}=0,\\ h_{9}(x)=x_{13}x_{30}-x_{14}x_{31}-x_{18}x_{32}=0,\\ h_{10}(x)=x_{13}x_{33}-x_{14}x_{34}-x_{18}x_{35}=0,\\ h_{11}(x)=x_{13}x_{36}-x_{14}x_{37}-x_{18}x_{35}=0,\\ h_{12}(x)=0.333x_{1}+x_{15}x_{31}-x_{5}x_{21}=0,\\ h_{13}(x)=0.333x_{1}+x_{15}x_{34}-x_{5}x_{24}=0,\\ h_{14}(x)=0.333x_{1}+x_{15}x_{37}-x_{5}x_{27}=0,\\ h_{15}(x)=0.333x_{2}+x_{10}x_{23}-x_{13}x_{30}=0,\\ h_{16}(x)=0.333x_{2}+x_{10}x_{26}-x_{13}x_{33}=0,\\ h_{17}(x)=0.333x_{2}+x_{10}x_{29}-x_{13}x_{36}=0,\\ h_{18}(x)=0.333x_{3}+x_{7}x_{22}+x_{11}x_{23}+x_{16}x_{31}+x_{19}x_{32}=30,\\ h_{19}(x)=0.333x_{3}+x_{7}x_{25}+x_{11}x_{26}+x_{16}x_{34}+x_{19}x_{35}=50,\\ h_{15}(x)=0.333x_{2}+x_{10}x_{23}-x_{13}x_{30}=0,\\ h_{16}(x)=0.333x_{2}+x_{10}x_{26}-x_{13}x_{33}=0,\\ h_{17}(x)=0.333x_{2}+x_{10}x_{29}-x_{13}x_{36}=0,\\ h_{18}(x)=0.333x_{3}+x_{7}x_{22}+x_{11}x_{23}+x_{16}x_{31}+x_{19}x_{32}=30,\\ h_{19}(x)=0.333x_{3}+x_{7}x_{25}+x_{11}x_{26}+x_{16}x_{34}+x_{19}x_{35}=50,\\ h_{20}(x)=0.333x_{3}+x_{7}x_{28}+x_{11}x_{29}+x_{16}x_{37}+x_{19}x_{38}=30,\\ h_{21}(x)=x_{21}+x_{24}+x_{27}=1,\\ h_{22}(x)=x_{22}+x_{25}+x_{28}=1,\\ h_{23}(x)=x_{23}+x_{26}+x_{29}=1,\\ h_{24}(x)=x_{30}+x_{33}+x_{36}=1,\\ h_{25}(x)=x_{31}+x_{34}+x_{37}=1,\\ h_{26}(x)=x_{32}+x_{35}+x_{38}=1,\\ h_{27}(x)=x_{25}=0,\\ h_{28}(x)=x_{28}=0,\\ h_{29}(x)=x_{23}=0,\\ h_{30}(x)=x_{37}=0,\\ h_{31}(x)=x_{32}=0,\\ h_{32}(x)=x_{35}=0, \end{array}$$

with bounds:

$$\begin{array}{l} 0\leq x_{1},x_{3},x_{8},x_{9},x_{5},x_{6},x_{14},x_{18},x_{10},x_{16},x_{13},x_{20}\leq 90,\\ 0\leq x_{2},x_{4},x_{7},x_{11},x_{12},x_{15},x_{17},x_{19}\leq 150,\\ 0\leq x_{21},x_{23},x_{24},x_{25},x_{27},x_{28}\leq 1,\\ 0\leq x_{22},x_{32},x_{34},x_{35},x_{37},x_{38}\leq 1.2,\\ 0\leq x_{26},x_{29},x_{30},x_{31},x_{33},x_{36}\leq 0.5. \end{array}$$

**Figure 17:**
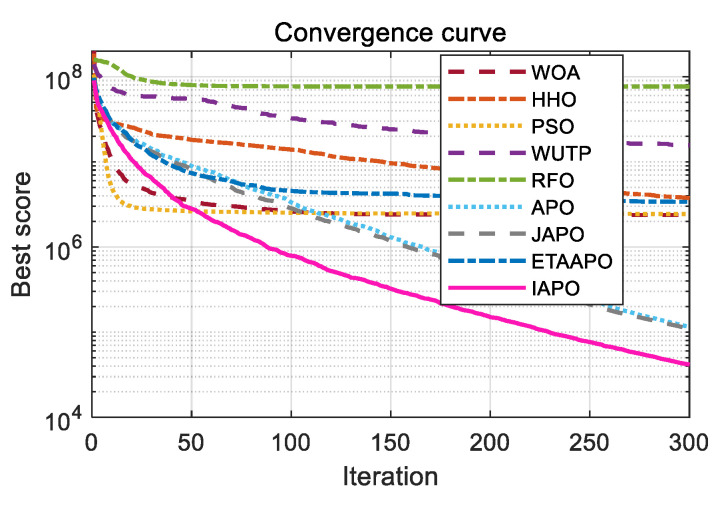
Convergence curves of the nine algorithms applied to the blending-pooling-separation problem.

The optimization results of IAPO and comparison algorithms on the blending-pooling-separation problem are summarized in Table 14 and Table 15. Table 14 reports the best, mean, and standard deviation values, while Table 15 provides the minimum cost and corresponding optimal variables for each algorithm. The results show that IAPO dominates the comparison, showing clear superiority across all three key metrics, attaining the lowest cost of 1.49E+04 with the optimal variable set: (32.5, 92.4, 59.9, 115.4, 28.2, 24.5, 6.8, 17.9, 3.8, 0.7, 2.2, 0.2, 80.7, 48.8, 15.4, 0.6, 32.3, 37.5, 28.9, 9.1, 0.6, 0.6, 0.1, 0.4, 0.5, 0.2, 0.6, 0.5, 0.5, 0.4, 0.4, 0.2, 0.4, 0.0, 0.9, 0.4, 0.4, 0.2). The convergence curves illustrated in Figure 17, confirm that IAPO converges most rapidly, with its curve consistently positioned at the lowest point. These findings demonstrate that IAPO is highly effective for this problem and successfully minimizes the total system cost.

To position this work within current research trends, this paper compares IAPO with several recently proposed algorithms through literature-based benchmarking, as shown in Table 16. IAPO demonstrates optimal performance across 20 benchmark functions, exhibits high applicability to engineering problems and robustness, and overall performance is relatively strong.

## 6. Conclusions and Expectations

This paper proposes an improved Arctic Puffin Optimization (IAPO) algorithm to address the shortcomings of slow initial convergence, susceptibility to local optima, and poor exploration-exploitation balance in the standard APO. The proposed IAPO algorithm integrates a lens imaging opposite learning mechanism with a dynamic differential evolution strategy. This paper selects three categories of metaheuristic algorithms for comparison: classical algorithms, recently proposed swarm intelligence algorithms, and improved variants of APO. Comprehensive experiments were conducted on 20 benchmark functions, along with the CEC 2019 and CEC 2022 test sets. The results show that IAPO achieves higher accuracy, faster convergence, and superior robustness, securing first-place average rankings of 1.35, 1.30, 1.25, and 1.08 on the 20 benchmark functions, CEC 2019, 10- and 20-dimensional CEC 2022 test sets, respectively. Additionally, through three engineering optimization simulation experiments, IAPO achieved optimal solutions of 5.2559 × 10^−4^, 1.09 × 10^3^, and 1.49 × 10^4^ for the respective engineering problems, ranking first in all cases. These results further validate the algorithm’s robust practical application value. However, the proposed IAPO also has limitations. Its performance was occasionally inferior to the standard APO on a small number of test functions. Moreover, while the adaptive strategy reduces the risk of premature convergence, it does not guarantee immunity from getting trapped in local optima in all scenarios.

Given that IAPO demonstrates superior overall performance on the test set and three engineering application problems despite suboptimal results on a few specific tests, the future work will focus on the following directions: (1) Applying IAPO to a wider range of practical engineering problems. (2) Replacing the maximum iteration limit with a maximum number of fitness evaluations as the stopping criterion to ensure experimental fairness. (3) Conducting comparative experiments between IAPO and more state-of-the-art algorithms on additional benchmark datasets. (4) Select appropriate strategies based on the characteristics of different optimization problems or combine them with other algorithms. (5) Extending the application of the IAPO algorithm to multi-objective optimization problems.

## Figures and Tables

**Table 1:** Set of 20 benchmark test functions.

Function	Name	Dimension	Range	Optimal Value	Function	Name	Dimension	Range	Optimal Value
F1	Sphere	30	[−100,100]	0	F11	Griewank	30	[−600,600]	0
F2	Schwefel 2.22	30	[−10,10]	0	F12	Penalized	30	[−50,50]	0
F3	Schwefel 1.2	30	[−100,100]	0	F13	Penalized2	30	[−50,50]	0
F4	Schwefel 2.21	30	[−100,100]	0	F14	Foxholes	2	[−65,65]	0.9980
F5	Rosenbrock	30	[−30,30]	0	F15	Kowalik’s	4	[−5,5]	0.0003
F6	Step	30	[−100,100]	0	F16	Six-Hump Camelback	2	[−5,5]	−1.3106
F7	Quartic	30	[−1.28,1.28]	0	F17	Hartman6	6	[0,1]	−3.3220
F8	Schwefel	30	[−500,500]	−12,569.5	F18	Shekel5	4	[0,10]	−10.1532
F9	Rastrigin	30	[−5.12,5.12]	0	F19	Shekel7	4	[0,10]	−10.4029
F10	Ackley	30	[−32,32]	0	F20	Shekel10	4	[0,10]	−10.5364

**Table 2:** CEC 2019 test function set.

Function	Name	Dimension	Range	Optimal Value
F1	Storn’s Chebyshev Polynomial Fitting Problem	9	[−8192,8192]	1
F2	Inverse Hilbert Matrix Problem	16	[−16,384,16,384]	1
F3	Lennard-Jones Minimum Energy Cluster	18	[−4,4]	1
F4	Rastrigin’s Function	10	[−100,100]	1
F5	Grienwank’s Function	10	[−100,100]	1
F6	Weierstrass Function	10	[−100,100]	1
F7	Modified Schaffer’s Function	10	[−100,100]	1
F8	Expanded Schaffer’s F6 Function	10	[−100,100]	1
F9	Happy Cat Function	10	[−100,100]	1
F10	Ackley Function	10	[−100,100]	1

**Table 3:** CEC 2022 test function set.

Function	Name	Dimension	Range	Optimal Value
F1	Shifted and Rotated Zakharov Function	10/20	[−100,100]	300
F2	Shifted and Rotated Rosenbrock’s Function	10/20	[−100,100]	400
F3	Shifted and Rotated Rastrigin’s Function	10/20	[−100,100]	600
F4	Shifted and Rotated Non-Continuous Rastrigin’s Function	10/20	[−100,100]	800
F5	Shifted and Rotated Levy Function	10/20	[−100,100]	900
F6	Hybrid Function 1 (N = 3)	10/20	[−100,100]	1800
F7	Hybrid Function 2 (N = 6)	10/20	[−100,100]	2000
F8	Hybrid Function 3 (N = 5)	10/20	[−100,100]	2200
F9	Composition Function 1 (N = 5)	10/20	[−100,100]	2300
F10	Composition Function 2 (N = 4)	10/20	[−100,100]	2400
F11	Composition Function 3 (N = 5)	10/20	[−100,100]	2600
F12	Composition Function 4 (N = 6)	10/20	[−100,100]	2700

**Table 4:** Comparative performance of nine Algorithms on 20 benchmark functions.

Function	Indicator	WOA	HHO	PSO	WUTP	RFO	APO	JAPO	ETAAPO	IAPO
F1	Best	1.02 × 10^−86^	6.25 × 10^−111^	4.30 × 10^3^	2.58 × 10^−1^	3.25 × 10^3^	1.23 × 10^−4^	8.66 × 10^−5^	6.55 × 10^−21^	**0**
	Mean	1.56 × 10^−69^	6.40 × 10^−96^	6.16 × 10^3^	8.82 × 10^−1^	5.83 × 10^3^	7.30 × 10^−4^	4.66 × 10^−4^	2.49 × 10^−17^	**0**
	Std	8.52 × 10^−69^	2.86 × 10^−95^	8.84 × 10^2^	6.66 × 10^−1^	1.79 × 10^3^	4.90 × 10^−4^	4.77 × 10^−4^	3.82 × 10^−17^	**0**
F2	Best	1.55 × 10^−58^	2.75 × 10^−61^	3.19 × 10^1^	2.64 × 10^−1^	2.19 × 10^1^	3.77 × 10^−3^	1.79 × 10^−3^	1.81 × 10^−12^	**0**
	Mean	4.61 × 10^−52^	4.58 × 10^−50^	3.57 × 10^1^	6.04 × 10^−1^	3.19 × 10^1^	8.05 × 10^−3^	6.29 × 10^−3^	1.50 × 10^−11^	**0**
	Std	1.95 × 10^−51^	2.19 × 10^−49^	2.48 × 10^0^	2.96 × 10^−1^	4.75 × 10^0^	2.20 × 10^−3^	3.43 × 10^−3^	1.20 × 10^−11^	**0**
F3	Best	3.13 × 10^4^	3.53 × 10^−99^	9.96 × 10^3^	1.11 × 10^4^	3.91 × 10^3^	1.92 × 10^−2^	5.22 × 10^−2^	3.07 × 10^−19^	**0**
	Mean	4.75 × 10^4^	1.63 × 10^−70^	1.45 × 10^4^	1.87 × 10^4^	8.64 × 10^3^	2.82 × 10^−1^	2.42 × 10^−1^	7.99 × 10^−15^	**0**
	Std	9.95 × 10^3^	8.92 × 10^−70^	2.63 × 10^3^	3.76 × 10^3^	4.22 × 10^3^	1.70 × 10^−1^	1.43 × 10^−1^	2.36 × 10^−14^	**0**
F4	Best	6.34 × 10^−2^	8.10 × 10^−57^	2.81 × 10^1^	4.87 × 10^0^	1.42 × 10^1^	3.23 × 10^−1^	3.48 × 10^−1^	3.01 × 10^−10^	**0**
	Mean	4.34 × 10^1^	2.06 × 10^−48^	3.17 × 10^1^	8.90 × 10^0^	2.80 × 10^1^	7.05 × 10^−1^	6.86 × 10^−1^	1.24 × 10^−7^	**0**
	Std	2.87 × 10^1^	1.12 × 10^−47^	2.53 × 10^0^	2.36 × 10^0^	5.78 × 10^0^	2.76 × 10^−1^	2.04 × 10^−1^	3.70 × 10^−7^	**0**
F5	Best	2.69 × 10^1^	4.25 × 10^−6^	1.66 × 10^6^	4.82 × 10^1^	4.71 × 10^5^	5.58 × 10^−1^	5.55 × 10^−1^	1.87 × 10^−2^	**1.48 × 10^−7^**
	Mean	2.81 × 10^1^	**8.56 × 10^−3^**	2.73 × 10^6^	2.37 × 10^2^	2.27 × 10^6^	2.68 × 10^1^	2.43 × 10^1^	1.28 × 10^0^	8.53 × 10^−1^
	Std	5.72 × 10^−1^	**1.41 × 10^−2^**	6.62 × 10^5^	1.83 × 10^2^	1.43 × 10^6^	4.98 × 10^0^	9.22 × 10^0^	1.70 × 10^0^	4.67 × 10^0^
F6	Best	4.05 × 10^−2^	5.77 × 10^−7^	3.94 × 10^3^	1.99 × 10^−1^	3.10 × 10^3^	5.06 × 10^−4^	1.28 × 10^−4^	1.10 × 10^−6^	**4.07 × 10^−14^**
	Mean	3.96 × 10^−1^	2.10 × 10^−4^	5.91 × 10^3^	7.42 × 10^−1^	6.17 × 10^3^	2.78 × 10^−3^	1.73 × 10^−3^	2.32 × 10^−2^	**4.56 × 10^−13^**
	Std	2.52 × 10^−1^	2.47 × 10^−4^	7.22 × 10^2^	4.12 × 10^−1^	1.96 × 10^3^	1.82 × 10^−3^	1.55 × 10^−3^	3.61 × 10^−2^	**3.00 × 10^−13^**
F7	Best	1.89 × 10^−4^	2.30 × 10^−6^	1.16 × 10^0^	1.40 × 10^−2^	2.42 × 10^−1^	9.59 × 10^−3^	1.68 × 10^−2^	2.39 × 10^−3^	**7.82 × 10^−6^**
	Mean	3.40 × 10^−3^	1.62 × 10^−4^	1.75 × 10^0^	4.03 × 10^−2^	1.08 × 10^0^	2.94 × 10^−2^	3.02 × 10^−2^	1.67 × 10^−2^	**6.09 × 10^−5^**
	Std	3.16 × 10^−3^	1.58 × 10^−4^	3.30 × 10^−1^	1.20 × 10^−2^	6.84 × 10^−1^	9.65 × 10^−3^	9.31 × 10^−3^	9.88 × 10^−3^	**4.97 × 10^−5^**
F8	Best	**−1.26 × 10^4^**	**−1.26 × 10^4^**	−7.81 × 10^3^	−5.15 × 10^3^	−5.63 × 10^3^	−8.70 × 10^3^	−9.01 × 10^3^	−7.49 × 10^3^	**−1.26 × 10^4^**
	Mean	−1.05 × 10^4^	−1.24 × 10^4^	−5.85 × 10^3^	−4.19 × 10^3^	−4.05 × 10^3^	−6.84 × 10^3^	−6.91 × 10^3^	−5.71 × 10^3^	**−1.26 × 10^4^**
	Std	1.79 × 10^3^	6.52 × 10^2^	7.23 × 10^2^	3.63 × 10^2^	7.00 × 10^2^	1.11 × 10^3^	1.12 × 10^3^	7.00 × 10^2^	**3.84 × 10^−2^**
F9	Best	**0**	**0**	2.08 × 10^2^	1.92 × 10^2^	1.34 × 10^2^	4.05 × 10^1^	3.91 × 10^1^	0.00 × 10^0^	**0**
	Mean	7.58 × 10^−15^	**0**	2.37 × 10^2^	2.16 × 10^2^	1.77 × 10^2^	1.00 × 10^2^	9.67 × 10^1^	7.70 × 10^−1^	**0**
	Std	1.97 × 10^−14^	**0**	1.27 × 10^1^	1.30 × 10^1^	2.69 × 10^1^	4.27 × 10^1^	4.01 × 10^1^	4.22 × 10^0^	**0**
F10	Best	**8.88 × 10^−16^**	**8.88 × 10^−16^**	1.28 × 10^1^	1.62 × 10^−1^	1.09 × 10^1^	3.49 × 10^−3^	3.11 × 10^−3^	7.16 × 10^−11^	**8.88 × 10^−16^**
	Mean	4.56 × 10^−15^	**8.88 × 10^−16^**	1.38 × 10^1^	4.42 × 10^−1^	1.32 × 10^1^	7.73 × 10^−3^	5.83 × 10^−3^	3.56 × 10^−10^	**8.88 × 10^−16^**
	Std	2.55 × 10^−15^	**0**	4.28 × 10^−1^	2.01 × 10^−1^	1.10 × 10^0^	3.54 × 10^−3^	2.20 × 10^−3^	3.36 × 10^−10^	**0**
F11	Best	**0**	**0**	4.22 × 10^1^	4.45 × 10^−1^	2.66 × 10^1^	4.74 × 10^−4^	1.96 × 10^−4^	0.00 × 10^0^	**0**
	Mean	1.21 × 10^−2^	**0**	5.74 × 10^1^	7.70 × 10^−1^	5.74 × 10^1^	6.96 × 10^−3^	5.95 × 10^−3^	2.01 × 10^−8^	**0**
	Std	6.63 × 10^−2^	**0**	8.21 × 10^0^	1.64 × 10^−1^	1.60 × 10^1^	8.21 × 10^−3^	8.27 × 10^−3^	1.10 × 10^−7^	**0**
F12	Best	4.49 × 10^−3^	5.85 × 10^−8^	1.98 × 10^2^	1.77 × 10^−2^	1.80 × 10^1^	6.06 × 10^−6^	1.28 × 10^−5^	2.62 × 10^−9^	**4.63 × 10^−15^**
	Mean	2.48 × 10^−2^	1.17 × 10^−5^	1.66 × 10^5^	2.92 × 10^−1^	6.30 × 10^4^	7.00 × 10^−3^	3.51 × 10^−3^	2.54 × 10^−2^	**2.27 × 10^−14^**
	Std	2.01 × 10^−2^	1.82 × 10^−5^	1.11 × 10^5^	3.72 × 10^−1^	2.12 × 10^5^	2.63 × 10^−2^	1.89 × 10^−2^	1.30 × 10^−1^	**1.61 × 10^−14^**
F13	Best	8.61 × 10^−2^	3.71 × 10^−7^	1.02 × 10^6^	1.87 × 10^−1^	1.17 × 10^5^	3.01 × 10^−4^	7.81 × 10^−5^	1.27 × 10^−5^	**5.03 × 10^−15^**
	Mean	4.61 × 10^−1^	8.21 × 10^−5^	3.23 × 10^6^	1.27 × 10^0^	2.90 × 10^6^	3.05 × 10^−3^	5.04 × 10^−3^	4.80 × 10^−2^	**1.11 × 10^−13^**
	Std	2.50 × 10^−1^	1.06 × 10^−4^	1.21 × 10^6^	1.67 × 10^0^	3.21 × 10^6^	4.35 × 10^−3^	6.62 × 10^−3^	4.74 × 10^−2^	**1.23 × 10^−13^**
F14	Best	**9.98 × 10^−1^**	**9.98 × 10^−1^**	**9.98 × 10^−1^**	**9.98 × 10^−1^**	**9.98 × 10^−1^**	**9.98 × 10^−1^**	**9.98 × 10^−1^**	**9.98 × 10^−1^**	**9.98 × 10^−1^**
	Mean	2.90 × 10^0^	5.01 × 10^0^	**9.98 × 10^−1^**	**9.98 × 10^−1^**	2.14 × 10^0^	**9.98 × 10^−1^**	1.06 × 10^0^	**9.98 × 10^−1^**	1.66 × 10^0^
	Std	2.95 × 10^0^	4.42 × 10^0^	3.61 × 10^−4^	1.54 × 10^−16^	2.62 × 10^0^	**0.00 × 10^0^**	3.62 × 10^−1^	**0.00 × 10^0^**	4.37 × 10^0^
F15	Best	3.08 × 10^−4^	3.08 × 10^−4^	7.05 × 10^−4^	6.16 × 10^−4^	**3.07 × 10^−4^**	**3.07 × 10^−4^**	**3.07 × 10^−4^**	**3.07 × 10^−4^**	**3.07 × 10^−4^**
	Mean	5.35 × 10^−4^	3.98 × 10^−4^	1.00 × 10^−3^	7.11 × 10^−4^	4.42 × 10^−3^	**3.07 × 10^−4^**	**3.07 × 10^−4^**	**3.07 × 10^−4^**	**3.07 × 10^−4^**
	Std	2.27 × 10^−4^	2.29 × 10^−4^	2.85 × 10^−4^	4.30 × 10^−5^	8.11 × 10^−3^	1.22 × 10^−19^	1.40 × 10^−19^	1.34 × 10^−19^	**1.11 × 10^−19^**
F16	Best	**−1.03 × 10^0^**	**−1.03 × 10^0^**	**−1.03 × 10^0^**	**−1.03 × 10^0^**	**−1.03 × 10^0^**	**−1.03 × 10^0^**	**−1.03 × 10^0^**	**−1.03 × 10^0^**	**−1.03 × 10^0^**
	Mean	**−1.03 × 10^0^**	**−1.03 × 10^0^**	**−1.03 × 10^0^**	**−1.03 × 10^0^**	**−1.03 × 10^0^**	**−1.03 × 10^0^**	**−1.03 × 10^0^**	**−1.03 × 10^0^**	**−1.03 × 10^0^**
	Std	1.48 × 10^−9^	1.85 × 10^−9^	4.73 × 10^−4^	1.57 × 10^−5^	**6.78 × 10^−16^**	**6.78 × 10^−16^**	**6.78 × 10^−16^**	**6.78 × 10^−16^**	**6.78 × 10^−16^**
F17	Best	**−3.32 × 10^0^**	−3.26 × 10^0^	−3.31 × 10^0^	**−3.32 × 10^0^**	**−3.32 × 10^0^**	**−3.32 × 10^0^**	**−3.32 × 10^0^**	**−3.32 × 10^0^**	**−3.32 × 10^0^**
	Mean	**−3.22 × 10^0^**	−3.15 × 10^0^	−3.24 × 10^0^	−3.24 × 10^0^	−3.27 × 10^0^	−3.31 × 10^0^	−3.31 × 10^0^	−3.30 × 10^0^	**−3.32 × 10^0^**
	Std	9.69 × 10^−2^	7.51 × 10^−2^	5.72 × 10^−2^	6.98 × 10^−2^	6.01 × 10^−2^	3.02 × 10^−2^	3.63 × 10^−2^	4.21 × 10^−2^	**1.34 × 10^−2^**
F18	Best	**−1.02 × 10^1^**	−1.01 × 10^1^	−9.89 × 10^0^	**−1.02 × 10^1^**	**−1.02 × 10^1^**	**−1.02 × 10^1^**	**−1.02 × 10^1^**	**−1.02 × 10^1^**	**−1.02 × 10^1^**
	Mean	−8.02 × 10^0^	−5.83 × 10^0^	−6.12 × 10^0^	−7.42 × 10^0^	−6.88 × 10^0^	**−1.02 × 10^1^**	**−1.02 × 10^1^**	**−1.02 × 10^1^**	**−1.02 × 10^1^**
	Std	2.89 × 10^0^	1.78 × 10^0^	2.78 × 10^0^	3.25 × 10^0^	3.03 × 10^0^	7.07 × 10^−15^	7.07 × 10^−15^	**6.63 × 10^−15^**	6.90 × 10^−15^
F19	Best	**−1.04 × 10^1^**	−5.09 × 10^0^	−9.92 × 10^0^	**−1.04 × 10^1^**	**−1.04 × 10^1^**	**−1.04 × 10^1^**	**−1.04 × 10^1^**	**−1.04 × 10^1^**	**−1.04 × 10^1^**
	Mean	−6.59 × 10^0^	−5.09 × 10^0^	−8.01 × 10^0^	**−1.04 × 10^1^**	−7.81 × 10^0^	**−1.04 × 10^1^**	−1.02 × 10^1^	**−1.04 × 10^1^**	**−1.04 × 10^1^**
	Std	3.56 × 10^0^	3.02 × 10^−3^	1.68 × 10^0^	1.65 × 10^−15^	3.31 × 10^0^	**9.90 × 10^−16^**	1.22 × 10^0^	1.40 × 10^−15^	1.23 × 10^−15^
F20	Best	**−1.05 × 10^1^**	**−1.05 × 10^1^**	−9.98 × 10^0^	**−1.05 × 10^1^**	**−1.05 × 10^1^**	**−1.05 × 10^1^**	**−1.05 × 10^1^**	**−1.05 × 10^1^**	**−1.05 × 10^1^**
	Mean	−6.97 × 10^0^	−5.39 × 10^0^	−8.56 × 10^0^	**−1.05 × 10^1^**	−8.33 × 10^0^	**−1.05 × 10^1^**	**−1.05 × 10^1^**	**−1.05 × 10^1^**	**−1.05 × 10^1^**
	Std	3.03 × 10^0^	1.47 × 10^0^	1.26 × 10^0^	3.09 × 10^−15^	3.47 × 10^0^	2.06 × 10^−15^	1.98 × 10^−15^	2.56 × 10^−15^	**1.81 × 10^−15^**

**Table 5:** Comparative performance of nine Algorithms on the CEC 2019 set.

Function	Indicator	WOA	HHO	PSO	WUTP	RFO	APO	JAPO	ETAAPO	IAPO
F1	Best	3.32 × 10^1^	**1.00 × 10^0^**	1.47 × 10^6^	2.14 × 10^6^	7.17 × 10^3^	**1.00 × 10^0^**	**1.00 × 10^0^**	**1.00 × 10^0^**	**1.00 × 10^0^**
	Mean	1.04 × 10^7^	**1.00 × 10^0^**	1.81 × 10^7^	6.64 × 10^6^	2.97 × 10^6^	3.55 × 10^0^	2.83 × 10^0^	2.65 × 10^1^	**1.00 × 10^0^**
	Std	1.41 × 10^7^	**0**	8.46 × 10^6^	2.98 × 10^6^	4.63 × 10^6^	6.65 × 10^0^	4.92 × 10^0^	3.66 × 10^1^	**0**
F2	Best	3.11 × 10^3^	4.60 × 10^0^	2.62 × 10^3^	3.76 × 10^3^	2.24 × 10^2^	3.98 × 10^0^	4.15 × 10^0^	4.36 × 10^0^	**3.93 × 10^0^**
	Mean	7.59 × 10^3^	4.96 × 10^0^	4.51 × 10^3^	5.70 × 10^3^	1.61 × 10^3^	5.21 × 10^0^	5.17 × 10^0^	4.68 × 10^0^	**4.38 × 10^0^**
	Std	2.90 × 10^3^	1.10 × 10^−1^	1.07 × 10^3^	9.01 × 10^2^	1.09 × 10^3^	9.22 × 10^−1^	7.18 × 10^−1^	2.25 × 10^−1^	**4.52 × 10^−1^**
F3	Best	1.29 × 10^0^	2.13 × 10^0^	6.99 × 10^0^	7.02 × 10^0^	3.70 × 10^0^	1.38 × 10^0^	**1.01 × 10^0^**	2.46 × 10^0^	1.19 × 10^0^
	Mean	5.62 × 10^0^	4.97 × 10^0^	8.49 × 10^0^	9.44 × 10^0^	6.93 × 10^0^	4.26 × 10^0^	3.73 × 10^0^	3.87 × 10^0^	**3.01 × 10^0^**
	Std	2.03 × 10^0^	1.31 × 10^0^	**6.67 × 10^−1^**	7.62 × 10^−1^	1.39 × 10^0^	1.94 × 10^0^	1.85 × 10^0^	8.60 × 10^−1^	1.02 × 10^0^
F4	Best	2.84 × 10^1^	2.62 × 10^1^	2.92 × 10^1^	3.46 × 10^1^	2.32 × 10^1^	5.97 × 10^0^	4.98 × 10^0^	1.43 × 10^1^	**1.00 × 10^0^**
	Mean	6.42 × 10^1^	5.22 × 10^1^	3.96 × 10^1^	4.90 × 10^1^	4.63 × 10^1^	1.06 × 10^1^	9.63 × 10^0^	3.03 × 10^1^	**8.48 × 10^0^**
	Std	1.71 × 10^1^	1.62 × 10^1^	6.19 × 10^0^	6.00 × 10^0^	1.34 × 10^1^	5.25 × 10^0^	2.77 × 10^0^	6.86 × 10^0^	**3.26 × 10^0^**
F5	Best	1.70 × 10^0^	1.60 × 10^0^	4.16 × 10^0^	1.36 × 10^0^	2.20 × 10^0^	**1.00 × 10^0^**	**1.00 × 10^0^**	2.50 × 10^0^	**1.00 × 10^0^**
	Mean	2.56 × 10^0^	2.04 × 10^0^	6.29 × 10^0^	1.83 × 10^0^	1.48 × 10^1^	1.07 × 10^0^	1.05 × 10^0^	8.27 × 10^0^	**1.04 × 10^0^**
	Std	7.74 × 10^−1^	3.31 × 10^−1^	1.23 × 10^0^	2.09 × 10^−1^	1.26 × 10^1^	5.53 × 10^−2^	**2.99 × 10^−2^**	4.14 × 10^0^	4.10 × 10^−2^
F6	Best	4.88 × 10^0^	3.08 × 10^0^	4.92 × 10^0^	1.03 × 10^0^	3.52 × 10^0^	**1.00 × 10^0^**	**1.00 × 10^0^**	2.15 × 10^0^	**1.00 × 10^0^**
	Mean	8.75 × 10^0^	8.17 × 10^0^	6.73 × 10^0^	1.54 × 10^0^	6.17 × 10^0^	**1.00 × 10^0^**	**1.00 × 10^0^**	4.58 × 10^0^	**1.00 × 10^0^**
	Std	2.12 × 10^0^	2.13 × 10^0^	6.73 × 10^−1^	5.59 × 10^−1^	1.27 × 10^0^	5.69 × 10^−4^	3.75 × 10^−4^	1.15 × 10^0^	**1.53 × 10^−8^**
F7	Best	7.81 × 10^2^	5.57 × 10^2^	1.34 × 10^3^	1.37 × 10^3^	9.53 × 10^2^	3.72 × 10^2^	5.02 × 10^1^	4.12 × 10^2^	**1.61 × 10^0^**
	Mean	1.37 × 10^3^	1.22 × 10^3^	1.67 × 10^3^	1.68 × 10^3^	1.48 × 10^3^	9.11 × 10^2^	6.86 × 10^2^	1.04 × 10^3^	**4.61 × 10^2^**
	Std	3.07 × 10^2^	2.95 × 10^2^	2.27 × 10^2^	**1.57 × 10^2^**	3.12 × 10^2^	3.44 × 10^2^	4.24 × 10^2^	2.48 × 10^2^	1.88 × 10^2^
F8	Best	4.02 × 10^0^	3.96 × 10^0^	3.87 × 10^0^	4.14 × 10^0^	3.79 × 10^0^	2.79 × 10^0^	3.12 × 10^0^	2.84 × 10^0^	**2.53 × 10^0^**
	Mean	4.72 × 10^0^	4.69 × 10^0^	4.41 × 10^0^	4.44 × 10^0^	4.60 × 10^0^	3.79 × 10^0^	3.82 × 10^0^	3.73 × 10^0^	**3.23 × 10^0^**
	Std	3.11 × 10^−1^	3.14 × 10^−1^	2.38 × 10^−1^	**1.43 × 10^−1^**	3.54 × 10^−1^	3.98 × 10^−1^	3.54 × 10^−1^	3.91 × 10^−1^	2.61 × 10^−1^
F9	Best	1.16 × 10^0^	1.16 × 10^0^	1.41 × 10^0^	1.24 × 10^0^	1.08 × 10^0^	1.10 × 10^0^	**1.04 × 10^0^**	**1.04 × 10^0^**	**1.04 × 10^0^**
	Mean	1.42 × 10^0^	1.46 × 10^0^	1.57 × 10^0^	1.32 × 10^0^	1.73 × 10^0^	1.17 × 10^0^	**1.16 × 10^0^**	**1.16 × 10^0^**	1.17 × 10^0^
	Std	1.70 × 10^−1^	2.22 × 10^−1^	7.93 × 10^−2^	5.11 × 10^−2^	9.09 × 10^−1^	**4.08 × 10^−2^**	5.56 × 10^−2^	7.19 × 10^−2^	5.34 × 10^−2^
F10	Best	2.10 × 10^1^	2.10 × 10^1^	2.12 × 10^1^	2.13 × 10^1^	2.12 × 10^1^	**1.00 × 10^0^**	**1.00 × 10^0^**	1.04 × 10^1^	**1.00 × 10^0^**
	Mean	2.13 × 10^1^	2.12 × 10^1^	2.15 × 10^1^	2.15 × 10^1^	2.14 × 10^1^	1.91 × 10^1^	1.66 × 10^1^	1.96 × 10^1^	**1.23 × 10^1^**
	Std	1.25 × 10^−1^	**1.05 × 10^−1^**	1.18 × 10^−1^	1.06 × 10^−1^	1.39 × 10^−1^	6.18 × 10^0^	8.77 × 10^0^	3.47 × 10^0^	9.49 × 10^0^

**Table 6:** Comparative performance of nine Algorithms on the 10-dimensional CEC 2022 set.

Function	Indicator	WOA	HHO	PSO	WUTP	RFO	APO	JAPO	ETAAPO	IAPO
F1	Best	5.29 × 10^3^	4.12 × 10^2^	8.96 × 10^2^	1.81 × 10^3^	3.93 × 10^2^	**3.00 × 10^2^**	**3.00 × 10^2^**	4.28 × 10^2^	**3.00 × 10^2^**
	Mean	2.64 × 10^4^	9.82 × 10^2^	1.68 × 10^3^	3.37 × 10^3^	4.38 × 10^3^	**3.00 × 10^2^**	**3.00 × 10^2^**	1.28 × 10^3^	**3.00 × 10^2^**
	Std	1.19 × 10^4^	4.05 × 10^2^	5.17 × 10^2^	1.03 × 10^3^	3.22 × 10^3^	3.17 × 10^−7^	2.29 × 10^−7^	5.53 × 10^2^	**9.89 × 10^−10^**
F2	Best	4.02 × 10^2^	**4.00 × 10^2^**	4.15 × 10^2^	4.68 × 10^2^	4.17 × 10^2^	**4.00 × 10^2^**	**4.00 × 10^2^**	4.01 × 10^2^	**4.00 × 10^2^**
	Mean	4.58 × 10^2^	4.41 × 10^2^	4.37 × 10^2^	5.00 × 10^2^	5.17 × 10^2^	**4.00 × 10^2^**	4.01 × 10^2^	4.69 × 10^2^	4.01 × 10^2^
	Std	6.68 × 10^1^	3.18 × 10^1^	2.97 × 10^1^	1.62 × 10^1^	1.34 × 10^2^	**1.06 × 10^0^**	2.82 × 10^0^	4.72 × 10^1^	2.47 × 10^0^
F3	Best	6.07 × 10^2^	6.15 × 10^2^	6.09 × 10^2^	6.02 × 10^2^	6.03 × 10^2^	**6.00 × 10^2^**	**6.00 × 10^2^**	6.06 × 10^2^	**6.00 × 10^2^**
	Mean	6.40 × 10^2^	6.38 × 10^2^	6.14 × 10^2^	6.06 × 10^2^	6.24 × 10^2^	**6.00 × 10^2^**	**6.00 × 10^2^**	6.10 × 10^2^	**6.00 × 10^2^**
	Std	1.40 × 10^1^	1.07 × 10^1^	3.05 × 10^0^	1.75 × 10^0^	9.79 × 10^0^	2.82 × 10^−4^	6.24 × 10^−5^	3.27 × 10^0^	**3.66 × 10^−14^**
F4	Best	8.22 × 10^2^	8.14 × 10^2^	8.24 × 10^2^	8.23 × 10^2^	8.14 × 10^2^	**8.03 × 10^2^**	**8.03 × 10^2^**	8.09 × 10^2^	8.04 × 10^2^
	Mean	8.44 × 10^2^	8.27 × 10^2^	8.37 × 10^2^	8.39 × 10^2^	8.33 × 10^2^	8.12 × 10^2^	**8.10 × 10^2^**	8.20 × 10^2^	8.17 × 10^2^
	Std	1.69 × 10^1^	6.17 × 10^0^	**4.77 × 10^0^**	5.74 × 10^0^	1.07 × 10^1^	5.79 × 10^0^	5.56 × 10^0^	5.50 × 10^0^	6.73 × 10^0^
F5	Best	9.44 × 10^2^	1.04 × 10^3^	9.44 × 10^2^	**9.00 × 10^2^**	9.23 × 10^2^	**9.00 × 10^2^**	**9.00 × 10^2^**	9.02 × 10^2^	**9.00 × 10^2^**
	Mean	1.51 × 10^3^	1.41 × 10^3^	1.00 × 10^3^	**9.00 × 10^2^**	1.17 × 10^3^	**9.00 × 10^2^**	**9.00 × 10^2^**	9.41 × 10^2^	**9.00 × 10^2^**
	Std	4.78 × 10^2^	1.39 × 10^2^	2.63 × 10^1^	6.01 × 10^−5^	1.72 × 10^2^	8.29 × 10^−2^	1.99 × 10^−10^	3.20 × 10^1^	**3.66 × 10^−14^**
F6	Best	2.48 × 10^3^	1.93 × 10^3^	9.93 × 10^4^	8.09 × 10^4^	1.83 × 10^3^	**1.80 × 10^3^**	**1.80 × 10^3^**	1.82 × 10^3^	**1.80 × 10^3^**
	Mean	5.62 × 10^3^	7.17 × 10^3^	2.11 × 10^6^	1.01 × 10^6^	2.00 × 10^3^	1.82 × 10^3^	1.82 × 10^3^	1.93 × 10^3^	**1.80 × 10^3^**
	Std	3.05 × 10^3^	5.52 × 10^3^	1.56 × 10^6^	8.38 × 10^5^	3.80 × 10^2^	1.24 × 10^1^	1.64 × 10^1^	3.55 × 10^2^	**2.41 × 10^0^**
F7	Best	2.02 × 10^3^	2.03 × 10^3^	2.04 × 10^3^	2.02 × 10^3^	2.03 × 10^3^	**2.00 × 10^3^**	**2.00 × 10^3^**	**2.00 × 10^3^**	**2.00 × 10^3^**
	Mean	2.08 × 10^3^	2.08 × 10^3^	2.06 × 10^3^	2.03 × 10^3^	2.06 × 10^3^	**2.00 × 10^3^**	**2.00 × 10^3^**	2.03 × 10^3^	**2.00 × 10^3^**
	Std	3.67 × 10^1^	3.48 × 10^1^	7.88 × 10^0^	6.17 × 10^0^	2.33 × 10^1^	2.83 × 10^0^	5.49 × 10^0^	8.33 × 10^0^	**1.06 × 10^−3^**
F8	Best	2.22 × 10^3^	2.21 × 10^3^	2.23 × 10^3^	2.23 × 10^3^	2.21 × 10^3^	**2.20 × 10^3^**	**2.20 × 10^3^**	**2.20 × 10^3^**	**2.20 × 10^3^**
	Mean	2.23 × 10^3^	2.23 × 10^3^	2.24 × 10^3^	2.23 × 10^3^	2.25 × 10^3^	**2.21 × 10^3^**	**2.21 × 10^3^**	2.22 × 10^3^	**2.21 × 10^3^**
	Std	9.00 × 10^0^	1.47 × 10^1^	3.09 × 10^1^	1.61 × 10^0^	4.89 × 10^1^	7.23 × 10^0^	7.47 × 10^0^	8.40 × 10^0^	**5.31 × 10^0^**
F9	Best	2.54 × 10^3^	2.54 × 10^3^	**2.53 × 10^3^**	2.54 × 10^3^	2.54 × 10^3^	**2.53 × 10^3^**	**2.53 × 10^3^**	**2.53 × 10^3^**	**2.53 × 10^3^**
	Mean	2.62 × 10^3^	2.61 × 10^3^	2.56 × 10^3^	2.55 × 10^3^	2.61 × 10^3^	**2.53 × 10^3^**	**2.53 × 10^3^**	2.58 × 10^3^	**2.53 × 10^3^**
	Std	5.31 × 10^1^	5.29 × 10^1^	5.01 × 10^1^	7.24 × 10^0^	4.58 × 10^1^	1.25 × 10^−5^	3.14 × 10^−6^	2.74 × 10^1^	**0.00 × 10^0^**
F10	Best	**2.50 × 10^3^**	**2.50 × 10^3^**	**2.50 × 10^3^**	**2.50 × 10^3^**	**2.50 × 10^3^**	**2.50 × 10^3^**	**2.50 × 10^3^**	**2.50 × 10^3^**	**2.50 × 10^3^**
	Mean	2.63 × 10^3^	2.65 × 10^3^	2.58 × 10^3^	**2.50 × 10^3^**	2.61 × 10^3^	**2.50 × 10^3^**	**2.50 × 10^3^**	**2.50 × 10^3^**	**2.50 × 10^3^**
	Std	2.68 × 10^2^	1.67 × 10^2^	7.36 × 10^1^	5.09 × 10^−1^	1.53 × 10^2^	7.38 × 10^−2^	6.13 × 10^−2^	1.73 × 10^0^	**4.72 × 10^−2^**
F11	Best	2.68 × 10^3^	2.61 × 10^3^	2.96 × 10^3^	**2.60 × 10^3^**	3.03 × 10^3^	**2.60 × 10^3^**	**2.60 × 10^3^**	2.72 × 10^3^	**2.60 × 10^3^**
	Mean	3.00 × 10^3^	2.83 × 10^3^	4.41 × 10^3^	2.85 × 10^3^	5.15 × 10^3^	**2.60 × 10^3^**	2.62 × 10^3^	2.79 × 10^3^	**2.60 × 10^3^**
	Std	1.48 × 10^2^	1.25 × 10^2^	6.74 × 10^2^	1.14 × 10^2^	1.99 × 10^3^	6.67 × 10^−6^	8.98 × 10^1^	6.45 × 10^1^	**4.22 × 10^−13^**
F12	Best	2.89 × 10^3^	2.87 × 10^3^	2.87 × 10^3^	2.88 × 10^3^	2.87 × 10^3^	**2.86 × 10^3^**	**2.86 × 10^3^**	2.87 × 10^3^	**2.86 × 10^3^**
	Mean	2.91 × 10^3^	2.91 × 10^3^	2.88 × 10^3^	2.89 × 10^3^	2.93 × 10^3^	**2.86 × 10^3^**	**2.86 × 10^3^**	2.88 × 10^3^	**2.86 × 10^3^**
	Std	5.04 × 10^1^	5.36 × 10^1^	4.67 × 10^1^	6.57 × 10^0^	4.14 × 10^1^	1.16 × 10^0^	8.35 × 10^−1^	1.28 × 10^1^	**1.20 × 10^−1^**

**Table 7:** Comparative performance of nine Algorithms on the 20-dimensional CEC 2022 set.

Function	Indicator	WOA	HHO	PSO	WUTP	RFO	APO	JAPO	ETAAPO	IAPO
F1	Best	1.77 × 10^4^	1.04 × 10^4^	9.65 × 10^3^	2.22 × 10^4^	1.57 × 10^4^	3.23 × 10^2^	3.31 × 10^2^	8.38 × 10^3^	**3.01 × 10^2^**
	Mean	3.90 × 10^4^	2.38 × 10^4^	1.43 × 10^4^	3.13 × 10^4^	2.81 × 10^4^	5.78 × 10^2^	5.79 × 10^2^	1.54 × 10^4^	**3.16 × 10^2^**
	Std	1.37 × 10^4^	7.12 × 10^3^	3.24 × 10^3^	6.30 × 10^3^	9.19 × 10^3^	2.73 × 10^2^	3.51 × 10^2^	3.93 × 10^3^	**2.08 × 10^1^**
F2	Best	4.83 × 10^2^	4.51 × 10^2^	4.97 × 10^2^	5.70 × 10^2^	6.54 × 10^2^	**4.45 × 10^2^**	**4.45 × 10^2^**	5.99 × 10^2^	**4.45 × 10^2^**
	Mean	6.22 × 10^2^	5.48 × 10^2^	5.48 × 10^2^	6.45 × 10^2^	1.38 × 10^3^	4.57 × 10^2^	4.54 × 10^2^	8.28 × 10^2^	**4.52 × 10^2^**
	Std	7.73 × 10^1^	4.62 × 10^1^	4.14 × 10^1^	4.86 × 10^1^	3.98 × 10^2^	1.14 × 10^1^	9.26 × 10^0^	1.28 × 10^2^	**7.57 × 10^0^**
F3	Best	6.37 × 10^2^	6.42 × 10^2^	6.20 × 10^2^	6.18 × 10^2^	6.34 × 10^2^	**6.00 × 10^2^**	**6.00 × 10^2^**	6.22 × 10^2^	**6.00 × 10^2^**
	Mean	6.70 × 10^2^	6.62 × 10^2^	6.29 × 10^2^	6.26 × 10^2^	6.60 × 10^2^	**6.00 × 10^2^**	**6.00 × 10^2^**	6.35 × 10^2^	**6.00 × 10^2^**
	Std	1.66 × 10^1^	8.07 × 10^0^	4.97 × 10^0^	3.56 × 10^0^	1.36 × 10^1^	1.10 × 10^−1^	1.11 × 10^−1^	6.94 × 10^0^	**3.97 × 10^−5^**
F4	Best	8.65 × 10^2^	8.56 × 10^2^	8.97 × 10^2^	9.02 × 10^2^	8.87 × 10^2^	8.25 × 10^2^	**8.18 × 10^2^**	8.77 × 10^2^	8.24 × 10^2^
	Mean	9.36 × 10^2^	8.87 × 10^2^	9.27 × 10^2^	9.33 × 10^2^	9.47 × 10^2^	8.60 × 10^2^	**8.44 × 10^2^**	9.03 × 10^2^	8.53 × 10^2^
	Std	3.50 × 10^1^	1.52 × 10^1^	1.27 × 10^1^	1.12 × 10^1^	2.41 × 10^1^	3.63 × 10^1^	2.76 × 10^1^	**1.37 × 10^1^**	2.23 × 10^1^
F5	Best	1.91 × 10^3^	2.32 × 10^3^	1.36 × 10^3^	9.01 × 10^2^	2.09 × 10^3^	**9.00 × 10^2^**	**9.00 × 10^2^**	1.23 × 10^3^	**9.00 × 10^2^**
	Mean	4.00 × 10^3^	3.03 × 10^3^	1.67 × 10^3^	9.38 × 10^2^	3.15 × 10^3^	9.01 × 10^2^	9.02 × 10^2^	1.62 × 10^3^	**9.00 × 10^2^**
	Std	1.18 × 10^3^	3.36 × 10^2^	1.54 × 10^2^	4.70 × 10^1^	7.43 × 10^2^	2.08 × 10^0^	3.16 × 10^0^	2.60 × 10^2^	**8.58 × 10^−2^**
F6	Best	5.10 × 10^5^	5.85 × 10^4^	3.44 × 10^7^	3.36 × 10^6^	7.74 × 10^3^	1.95 × 10^3^	**1.89 × 10^3^**	3.89 × 10^3^	**1.89 × 10^3^**
	Mean	6.63 × 10^6^	2.47 × 10^5^	1.15 × 10^8^	2.75 × 10^7^	2.15 × 10^8^	3.32 × 10^3^	3.31 × 10^3^	2.36 × 10^7^	**2.62 × 10^3^**
	Std	8.80 × 10^6^	1.14 × 10^5^	4.76 × 10^7^	3.84 × 10^7^	4.43 × 10^8^	2.18 × 10^3^	1.81 × 10^3^	4.45 × 10^7^	**9.44 × 10^2^**
F7	Best	2.13 × 10^3^	2.10 × 10^3^	2.11 × 10^3^	2.10 × 10^3^	2.07 × 10^3^	**2.01 × 10^3^**	2.02 × 10^3^	2.04 × 10^3^	**2.01 × 10^3^**
	Mean	2.23 × 10^3^	2.18 × 10^3^	2.14 × 10^3^	2.13 × 10^3^	2.17 × 10^3^	2.04 × 10^3^	2.04 × 10^3^	2.08 × 10^3^	**2.03 × 10^3^**
	Std	5.30 × 10^1^	4.40 × 10^1^	3.10 × 10^1^	1.32 × 10^1^	7.11 × 10^1^	1.14 × 10^1^	1.06 × 10^1^	2.30 × 10^1^	**3.53 × 10^0^**
F8	Best	2.23 × 10^3^	2.24 × 10^3^	2.24 × 10^3^	2.25 × 10^3^	2.23 × 10^3^	**2.22 × 10^3^**	**2.22 × 10^3^**	2.23 × 10^3^	**2.22 × 10^3^**
	Mean	2.31 × 10^3^	2.28 × 10^3^	2.31 × 10^3^	2.26 × 10^3^	2.32 × 10^3^	**2.23 × 10^3^**	**2.23 × 10^3^**	**2.23 × 10^3^**	**2.23 × 10^3^**
	Std	8.20 × 10^1^	7.64 × 10^1^	5.85 × 10^1^	1.33 × 10^1^	1.29 × 10^2^	1.76 × 10^0^	2.05 × 10^0^	2.32 × 10^1^	**1.09 × 10^0^**
F9	Best	2.51 × 10^3^	2.49 × 10^3^	2.49 × 10^3^	2.51 × 10^3^	2.63 × 10^3^	**2.48 × 10^3^**	**2.48 × 10^3^**	2.54 × 10^3^	**2.48 × 10^3^**
	Mean	2.60 × 10^3^	2.53 × 10^3^	2.53 × 10^3^	2.56 × 10^3^	2.80 × 10^3^	**2.48 × 10^3^**	**2.48 × 10^3^**	2.62 × 10^3^	**2.48 × 10^3^**
	Std	5.59 × 10^1^	3.53 × 10^1^	5.43 × 10^1^	2.30 × 10^1^	1.19 × 10^2^	4.25 × 10^−1^	6.70 × 10^−1^	5.20 × 10^1^	**3.66 × 10^−4^**
F10	Best	**2.50 × 10^3^**	**2.50 × 10^3^**	2.51 × 10^3^	2.51 × 10^3^	2.62 × 10^3^	**2.50 × 10^3^**	**2.50 × 10^3^**	2.52 × 10^3^	**2.50 × 10^3^**
	Mean	5.03 × 10^3^	4.33 × 10^3^	3.87 × 10^3^	2.52 × 10^3^	5.27 × 10^3^	**2.50 × 10^3^**	**2.50 × 10^3^**	2.57 × 10^3^	**2.50 × 10^3^**
	Std	1.24 × 10^3^	8.68 × 10^2^	1.93 × 10^3^	5.63 × 10^0^	1.59 × 10^3^	1.63 × 10^−1^	1.37 × 10^−1^	1.07 × 10^2^	**9.71 × 10^−2^**
F11	Best	3.52 × 10^3^	3.04 × 10^3^	1.48 × 10^4^	2.92 × 10^3^	1.35 × 10^4^	2.90 × 10^3^	2.90 × 10^3^	3.94 × 10^3^	**2.60 × 10^3^**
	Mean	3.98 × 10^3^	3.74 × 10^3^	1.93 × 10^4^	2.94 × 10^3^	2.62 × 10^4^	**2.90 × 10^3^**	**2.90 × 10^3^**	5.51 × 10^3^	2.91 × 10^3^
	Std	2.72 × 10^2^	6.53 × 10^2^	2.26 × 10^3^	1.22 × 10^1^	1.01 × 10^4^	3.28 × 10^−1^	**2.93 × 10^−1^**	6.20 × 10^2^	6.91 × 10^1^
F12	Best	2.97 × 10^3^	3.00 × 10^3^	2.95 × 10^3^	3.10 × 10^3^	3.15 × 10^3^	2.94 × 10^3^	2.94 × 10^3^	3.07 × 10^3^	**2.93 × 10^3^**
	Mean	3.08 × 10^3^	3.20 × 10^3^	3.01 × 10^3^	3.20 × 10^3^	3.51 × 10^3^	2.96 × 10^3^	2.95 × 10^3^	3.20 × 10^3^	**2.94 × 10^3^**
	Std	8.97 × 10^1^	1.86 × 10^2^	5.31 × 10^1^	4.93 × 10^1^	2.50 × 10^2^	1.37 × 10^1^	1.17 × 10^1^	9.99 × 10^1^	**5.37 × 10^0^**

**Table 8:** Wilcoxon Rank-Sum Test Statistics.

IAPO vs.	Algorithm	20 Benchmark Functions	CEC 2019 Test Set	CEC 2022 Test Set (D = 10)	CEC 2022 Test Set (D = 20)
Wilcoxon Rank-Sum Test (+/=/−)	WOA	17/3/0	10/0/0	12/0/0	12/0/0
	HHO	20/0/0	9/0/1	11/1/0	12/0/0
	PSO	19/1/0	10/0/0	12/0/0	12/0/0
	WUTP	20/0/0	10/0/0	12/0/0	10/2/0
	RFO	17/3/0	9/0/1	12/0/0	12/0/0
	APO	16/3/1	7/3/0	9/2/1	12/0/0
	JAPO	16/3/1	7/3/0	11/0/1	10/2/0
	ETAAPO	17/2/1	8/2/0	12/0/0	12/0/0
	Total	142/15/3	70/8/2	91/3/2	94/2/0

**Table 9:** Friedman test results.

/	20 Benchmark Functions		CEC 2019 Test Set		CEC 2022 (D = 10) Test Set		CEC 2022 (D = 20) Test Set	
Algorithm	Average Rank	Rank	Average Rank	Rank	Average Rank	Rank	Average Rank	Rank
WOA	4.19	6	7.42	9	7.82	9	7.49	8
HHO	4.05	5	4.79	6	5.70	6	5.28	5
PSO	4.77	7	4.49	4	4.54	4	3.92	4
WUTP	5.85	8	7.36	8	6.76	7	6.57	7
RFO	6.37	9	7.32	7	7.47	8	8.23	9
APO	3.56	4	3.43	3	2.78	3	2.81	3
JAPO	3.29	3	3.01	2	2.49	2	2.73	2
ETAAPO	2.77	2	4.64	5	5.54	5	6.48	6
IAPO	**1.15**	**1**	**2.02**	**1**	**1.50**	**1**	**1.48**	**1**

**Table 10:** Optimization results for the planetary gear train design problem.

Indicator	WOA	HHO	PSO	WUTP	RFO	APO	JAPO	ETAAPO	IAPO
Best	5.2735 × 10^−1^	5.2577 × 10^−1^	5.3706 × 10^−1^	5.6300 × 10^−1^	5.3236 × 10^−1^	5.2577 × 10^−1^	5.2577 × 10^−1^	5.2577 × 10^−1^	**5.2559** × **10^−^^1^**
Mean	6.0987 × 10^−1^	5.3638 × 10^−1^	6.4595 × 10^−1^	7.4550 × 10^−1^	1.2129 × 10^0^	5.2826 × 10^−1^	5.2875 × 10^−1^	5.3147 × 10^−1^	**5.2790** × **10^−^^1^**
Std	9.4882 × 10^−2^	1.0106 × 10^−2^	2.4300 × 10^−1^	1.2548 × 10^−1^	6.9410 × 10^−1^	**1.8985** × **10^−^^3^**	3.4490 × 10^−3^	5.0353 × 10^−3^	2.0440 × 10^−3^
Rank	6	5	7	8	9	4	2	3	**1**

**Table 11:** Independent variables corresponding to experimental results.

Algorithm	Norm									Best
	*x*1	*x*2	*x*3	*x*4	*x*5	*x*6	*x*7	*x*8	*x*9	
WOA	42	25	20	24	25	87	1.5781	1.9996	1.4096	5.2735 × 10^−1^
HHO	42	30	24	24	23	87	1.1278	2.1629	1.2861	5.2577 × 10^−1^
PSO	38	33	40	33	14	120	2.0000	1.0000	1.1563	5.3706 × 10^−1^
WUTP	35	24	22	24	25	87	3.4818	1.6355	1.1661	5.6300 × 10^−1^
RFO	51	26	20	29	36	107	2.2973	1.4142	1.0956	5.3236 × 10^−1^
APO	37	22	20	24	25	87	3.9063	2.8195	1.3820	5.2577 × 10^−1^
JAPO	35	26	25	24	21	87	1.8549	1.5882	1.2334	5.2577 × 10^−1^
ETAAPO	35	26	25	24	25	87	2.9835	2.7809	1.1835	5.2577 × 10^−1^
IAPO	35	26	25	24	20	87	1.5461	2.0606	1.4739	5.2559 × 10^−1^

**Table 12:** Optimization results for the heat exchanger network design problem.

Indicator	WOA	HHO	PSO	WUTP	RFO	APO	JAPO	ETAAPO	IAPO
Best	1.31 × 10^11^	7.96 × 10^9^	4.98 × 10^9^	8.98 × 10^13^	9.39 × 10^8^	3.23 × 10^6^	2.79 × 10^5^	1.40 × 10^8^	**1.09** × **10^3^**
Mean	2.45 × 10^14^	3.89 × 10^14^	1.34 × 10^14^	3.19 × 10^14^	2.27 × 10^14^	1.64 × 10^9^	2.95 × 10^8^	7.27 × 10^12^	**5.35** × **10^6^**
Std	3.26 × 10^14^	4.11 × 10^14^	4.07 × 10^14^	1.23 × 10^14^	4.15 × 10^14^	5.05 × 10^9^	5.66 × 10^8^	2.69 × 10^13^	**2.75** × **10^7^**
Rank	5	8	6	9	7	3	2	4	**1**

**Table 13:** Independent variables corresponding to experimental results.

Algorithm	Norm									Best
	*x1*	*x2*	*x3*	*x4*	*x5*	*x6*	*x7*	*x8*	*x9*	
WOA	4.362	155.332	99.244	1.057	1,984,877.272	63.785	100.858	599.977	701.717	1.31 × 10^11^
HHO	0.266	21.099	18.153	17.008	1,999,810.207	473.322	100.042	599.943	700.098	7.96 × 10^9^
PSO	9.243	80.114	83.735	0.000	2,000,000.000	124.693	100.000	600.000	699.916	4.98 × 10^9^
WUTP	4.919	51.101	14.953	88.623	1,912,821.010	169.528	118.924	595.857	704.465	8.98 × 10^13^
RFO	0.022	24.309	40.891	157.277	1,999210.439	411.170	100.060	599.988	700.044	9.39 × 10^8^
APO	3.877	104.385	67.432	0.092	1,999,897.728	95.794	100.012	599.994	700.008	3.23 × 10^6^
JAPO	0.045	30.486	61.723	6.930	1,999,935.023	328.011	100.006	599.993	700.006	2.79 × 10^5^
ETAAPO	0.235	190.614	63.126	4.063	1,999,803.081	52.459	100.024	599.968	700.034	1.40 × 10^8^
IAPO	0.809	98.130	78.244	0.483	1,999,921.277	101.902	100.008	599.992	700.008	1.09 × 10^3^

**Table 14:** Optimization results for the blending-pooling-separation problem.

Indicator	WOA	HHO	PSO	WUTP	RFO	APO	JAPO	ETAAPO	IAPO
Best	4.19 × 10^5^	4.73 × 10^5^	2.43 × 10^5^	1.01 × 10^7^	1.46 × 10^7^	5.92 × 10^4^	3.26 × 10^4^	1.71 × 10^6^	**1.49** × **10^4^**
Mean	2.37 × 10^6^	3.75 × 10^6^	2.44 × 10^6^	1.56 × 10^7^	7.64 × 10^7^	1.15 × 10^5^	1.10 × 10^5^	3.39 × 10^6^	**4.15** × **10^4^**
Std	2.48 × 10^6^	2.73 × 10^6^	2.73 × 10^6^	2.98 × 10^6^	3.43 × 10^7^	3.62 × 10^4^	3.48 × 10^4^	1.08 × 10^6^	**2.33** × **10^4^**
Rank	6	5	4	8	9	3	2	7	**1**

**Table 15:** Independent variables corresponding to experimental results.

Algorithm	Norm																		
	*x*1	*x*2	*x*3	*x*4	*x*5	*x*6	*x*7	*x*8	*x*9	*x*10	*x*11	*x*12	*x*13	*x*14	*x*15	*x*16	*x*17	*x*18	*x*19
WOA	0	54	90	150	0	3	0	0.1	1.4	0.0	1.6	0.0	34	0	0.1	5	0	23	22
HHO	24	31	90	150	4	4	11	0.2	2.8	0.0	5.3	6.2	15	18	2.4	0	14	13	1
PSO	0	150	0	150	0	90	89	0.0	0.0	0.0	0.0	0.0	90	90	0.0	87	0	18	18
WUTP	18	94	63	83	22	51	2	14.2	4.8	7.9	15.7	22.2	56	26	0.7	19	36	14	30
RFO	66	79	43	111	48	2	2	0.2	39.9	68.5	11.5	4.5	30	72	26.8	33	45	54	47
APO	43	71	51	135	25	28	19	9.1	3.0	0.6	1.2	1.0	75	35	2.0	27	5	28	2
JAPO	54	79	35	133	45	33	13	19.9	2.6	0.3	1.1	1.1	72	51	19.0	12	20	39	38
ETAAPO	67	81	81	60	29	31	2	27.5	0.3	3.6	0.7	11.3	54	61	6.6	10	41	39	46
IAPO	33	92	60	115	28	24	7	17.9	3.8	0.7	2.2	0.2	81	49	15.4	1	32	38	29
**Norm**																			**Best**
***x*** **20**	***x*** **21**	***x*** **22**	***x*** **23**	***x*** **24**	***x*** **25**	***x*** **26**	***x*** **27**	***x*** **28**	***x*** **29**	***x*** **30**	***x*** **31**	***x*** **32**	***x*** **33**	***x*** **34**	***x*** **35**	***x*** **36**	***x*** **37**	***x*** **38**	
0	0.0	1.2	0.0	0.1	0.0	0.3	0.0	0.5	0.1	0.0	0.5	0.0	0.5	0.0	0.8	0.5	0.7	0.0	4.19 × 10^5^
17	1.0	0.0	1.0	0.2	1.0	0.5	0.1	0.0	0.4	0.4	0.0	0.1	0.5	0.1	0.2	0.5	0.2	0.0	4.73 × 10^5^
1	0.2	0.0	0.4	1.0	0.0	0.4	1.0	0.0	0.5	0.5	0.4	0.0	0.5	0.3	0.8	0.5	0.4	0.0	2.43 × 10^5^
15	0.4	0.4	0.3	0.4	0.3	0.3	0.5	0.6	0.4	0.3	0.1	0.4	0.3	0.7	0.4	0.5	0.8	0.6	1.01 × 10^7^
43	0.5	0.2	0.2	0.5	1.0	0.5	0.7	0.2	0.2	0.2	0.2	0.2	0.3	0.3	0.1	0.2	0.4	0.5	1.46 × 10^7^
27	0.5	0.4	0.5	0.7	0.7	0.4	0.5	0.3	0.2	0.3	0.1	0.8	0.3	0.6	0.2	0.3	0.3	0.2	5.92 × 10^4^
2	0.5	0.7	0.0	0.4	0.6	0.1	0.5	0.7	0.4	0.3	0.3	0.2	0.4	0.0	0.7	0.4	0.3	0.2	3.26 × 10^4^
0	0.4	0.3	0.7	0.4	0.7	0.0	0.8	0.8	0.2	0.5	0.3	0.1	0.3	0.4	0.5	0.4	0.2	0.1	1.71 × 10^6^
9	0.6	0.6	0.1	0.4	0.5	0.2	0.6	0.5	0.5	0.4	0.4	0.2	0.4	0.0	0.9	0.4	0.4	0.2	1.49 × 10^4^

**Table 16:** Performance comparison of IAPO and the latest algorithm on 20 benchmark functions.

Algorithm	Publication Year	Number of Optimal Solutions (20 Functions)	Engineering Applicability	Robustness
IAPO	This Experiment	17 (Dim = 30)	Excellent Performance	Strong (Stable performance across different test sets)
DOA [41]	2025	9 (Dim = 30)	Moderate Performance	Poor performance at CEC2022 set
E2 [42]	2025	Not Tested	Top-ranked Performance	Not tested on the dataset
CCO [43]	2025	18 (Dim = 10)	Not Tested	Strong (Demonstrates superior performance across diverse test sets)
PPO [44]	2025	Not Tested	Superior Performance	Average (Average performance at CEC2022 set)

## Data Availability

The data that support the findings of this study are available from the corresponding author upon request. There are no restrictions on data availability.
